# Development of NanoLuc-PEST expressing *Leishmania mexicana* as a new drug discovery tool for axenic- and intramacrophage-based assays

**DOI:** 10.1371/journal.pntd.0006639

**Published:** 2018-07-12

**Authors:** Sarah L. Berry, Hamza Hameed, Anna Thomason, Marissa L. Maciej-Hulme, Somaia Saif Abou-Akkada, Paul Horrocks, Helen P. Price

**Affiliations:** 1 Centre for Applied Entomology and Parasitology, School of Life Sciences, Keele University, Newcastle-under-Lyme, Staffordshire, United Kingdom; 2 Institute for Science and Technology in Medicine, Keele University, Newcastle-under-Lyme, Staffordshire, United Kingdom; 3 Current address: School of Environment and Life Sciences, University of Salford, Salford, United Kingdom; 4 Radboud University Medical Center, Department of Nephrology, Geert Grooteplein 10, GA Nijmegan, The Netherlands; 5 Faculty of Veterinary Medicine, Alexandria University, Alexandria, Egypt; McGill University, CANADA

## Abstract

The protozoan parasite *Leishmania* causes leishmaniasis; a spectrum of diseases of which there are an estimated 1 million new cases each year. Current treatments are toxic, expensive, difficult to administer, and resistance to them is emerging. New therapeutics are urgently needed, however, screening the infective amastigote form of the parasite is challenging. Only certain species can be differentiated into axenic amastigotes, and compound activity against these does not always correlate with efficacy against the parasite in its intracellular niche. Methods used to assess compound efficacy on intracellular amastigotes often rely on microscopy-based assays. These are laborious, require specialist equipment and can only determine parasite burden, not parasite viability. We have addressed this clear need in the anti-leishmanial drug discovery process by producing a transgenic *L*. *mexicana* cell line that expresses the luciferase NanoLuc-PEST. We tested the sensitivity and versatility of this transgenic strain, in comparison with strains expressing NanoLuc and the red-shifted firefly luciferase. We then compared the NanoLuc-PEST luciferase to the current methods in both axenic and intramacrophage amastigotes following treatment with a supralethal dose of Amphotericin B. NanoLuc-PEST was a more dynamic indicator of cell viability due to its high turnover rate and high signal:background ratio. This, coupled with its sensitivity in the intramacrophage assay, led us to validate the NanoLuc-PEST expressing cell line using the MMV Pathogen Box in a two-step process: i) identify hits against axenic amastigotes, ii) screen these hits using our bioluminescence-based intramacrophage assay. The data obtained from this highlights the potential of compounds active against *M*. *tuberculosis* to be re-purposed for use against *Leishmania*. Our transgenic *L*. *mexicana* cell line is therefore a highly sensitive and dynamic system suitable for *Leishmania* drug discovery in axenic and intramacrophage amastigote models.

## Author summary

The protozoan parasite *Leishmania* causes a spectrum of diseases collectively known as leishmaniasis. The parasite is transmitted to humans by the bite of its vector, the sand fly, following which the parasite invades host white blood cells, particularly macrophages. Leishmaniasis is classified as a neglected tropical disease, and is endemic in 97 countries. Symptoms of the disease depend on the species of *Leishmania*. These include skin lesions, destruction of the mucosal membranes, and the visceral form which is usually fatal if untreated. Current therapeutic options for leishmaniasis have a number of associated problems that include toxicity, the development of drug resistance and poor patient compliance due to lengthy and painful treatment regimens. New therapeutics are therefore urgently needed. The ability to screen potential drug candidates requires robust screening assays. Currently, screening the intracellular parasite relies on microscopy-based techniques that require expensive equipment, are time consuming and only detect parasite burden, not viability. By using a transgenic cell line that expresses the NanoLuc-PEST luciferase, we show that we have a parasite-specific viability marker that can be used to measure the efficacy of compounds against the intracellular parasite. We validate the potential of this cell line by screening the MMV Pathogen Box.

## Introduction

The leishmaniases are a spectrum of diseases caused by infection with protozoan pathogens of the *Leishmania* genus, with an estimated 1 million new cases per annum. [1] *Leishmania* parasites are transmitted to a mammalian host via the bite of an infected sand fly. Highly motile metacyclic promastigotes invade host macrophages and differentiate into the amastigote form, which is highly adapted for intracellular survival. [2] Current treatments for leishmaniasis (reviewed in [3, 4]) are unsatisfactory due to high associated toxicity, cost, complex administration and the emergence of resistant strains. Efforts have greatly increased over the last decade to identify novel compounds with anti-leishmanial properties, or to repurpose existing drugs to widen the therapeutic options for this disease. The Drugs for Neglected Diseases *initiative* (DND*i*) was set up to identify potential lead compounds. This has yielded success with the identification of three new chemical series that display considerable anti-leishmanial potential. [5]

Efficient compound screening requires robust, sensitive and reproducible assays that are suitable for high throughput application. These assays primarily fall into two categories: target directed screening, and phenotypic screening. Recent advances have been made to bridge this gap using yeast-based systems, which express leishmanial target proteins for screening. [6–8] The main argument for phenotypic assays is that they directly measure compound activity against the target cell. This tends to be determined using either a colourmetric or fluorescent regent that measures metabolism (e.g. MTT [9] or AlamarBlue [10]), or using a fluorescent reporter molecule such as GFP [11–13] or mCherry. [14] The metabolism-based reagents are useful for studying the parasite alone in either its promastigote or axenic amastigote form, but they cannot distinguish between the parasite and host cells in an *in vitro* cell infection model. Parasite-specific fluorescent reporter molecules show the presence and localisation of parasites; however the assays used tend to look for the presence of the fluorescent parasite (either by microscopy or flow cytometry), not parasite viability.

Screening for novel anti-leishmanial compounds using an intramacrophage model is more relevant, and is likely to provide a better translation of hits. This is because the model incorporates both the multiple membranes that the compound must traverse to reach the amastigote, and the environment within the parasitophorous vacuole. The current methods for assessing efficacy against the amastigote in its intramacrophage niche involve either flow cytometric or microscopy-based techniques. These can be scaled up to a high content screening system, [15] but this requires the use of specialist and expensive equipment. In addition, these methods can only detect parasite burden, not viability. One technology that can overcome this hurdle is the use of bioluminescence. Bioluminescence offers a dynamic method for determining both the viability and location of the tagged cell, and has been applied to a number of infectious disease models. [16–18] This technology utilises transgenic pathogens that express one or more luciferases; a diverse group of enzymes that have the ability to generate light in the presence of a specific substrate. In *Plasmodium falciparum*, luciferases have proven to be robust and sensitive reporters in drug screens. [16, 19–22] For kinetoplastid research, luciferases from the North American firefly (*Photinus pyralis*) and the sea pansy (*Renilla reniformis*) have been used for drug screening [23–25] and *in vivo* studies. [26–29] A red-shifted variant of the firefly luciferase (PRE9) shows significantly improved sensitivity in *Trypanosoma brucei* infection in animal models, and has proven to be a powerful technique for detecting low numbers of parasites in the brain. [30]

Whilst luciferases derived from *P*. *pyralis* and *R*. *reniformis* are the most commonly used, other luciferases with differing properties are now being explored. A luciferase isolated from the deep sea shrimp (*Oplophorus gracilirostris*), known as NanoLuc, is a relatively small (19 kDa, compared to 61 kDa and 36 kDa for *P*. *pyralis* and *R*. *reniformis* respectively) and very stable enzyme that produces a high intensity, glow-type bioluminescence. [31] A modified form of the enzyme, NanoLuc-PEST (23 kDa), retains high enzymatic activity but has a reduced intracellular half-life due to fusion of a PEST sequence, which marks the molecule for rapid degradation. [31] NanoLuc has been successfully expressed in *Plasmodium falciparum* [32] but there are no reports to date describing expression of this reporter (or the PEST-fusion derivative) in kinetoplastids.

New molecular tools, such as the NanoLuc-PEST enzyme, are being used to improve compound screening to aid drug development. [33] This study focuses on the use of transgenic parasites expressing this enzyme, in both axenic- and intramacrophage-based assays. This new method, which specifically detects amastigote viability within the macrophage, will help bridge the gap between axenic and *in vivo* testing, in a manner that is time efficient and scalable to high-throughput systems.

## Methods

### Cell culture

*L*. *mexicana* strain MNYC/BZ/62/M379 was maintained *in vitro* in the procyclic promastigote stage by culture at 26°C in Schneider’s medium (Gibco) pH 7.0 containing 10% FBS (Gibco), 100 U/mL penicillin (Lonza) and 100 μg/mL streptomycin (Lonza). Differentiation to the axenic amastigote stage was performed as described previously. [34] Briefly, axenic amastigotes were cultivated at 32°C in Schneider’s medium pH 5.5 supplemented with 10% FBS, 100 U/mL penicillin and 100 μg/mL streptomycin (complete Schneider’s media pH 5.5). The human monocyte cell line THP-1 [35] was maintained *in vitro* by culturing at 37°C with 5% CO_2_ in Dutch modified RPMI-1640 (Gibco) containing 10% FBS and 2 mM L-glutamine (Gibco) (complete RPMI media). Differentiation of THP-1 cells into macrophages was performed by seeding 2.5 x10^5^ cells/mL in complete RPMI media, supplemented with 20 ng/mL phorbol 12-myristate 13-acetate (PMA). [36] Cells were incubated at 37°C with 5% CO_2_ for 24 hours.

### Generation of plasmid constructs and *L*. *mexicana* transfection

NanoLuc, NanoLuc-PEST and red-shifted firefly luciferase (PRE9) open reading frames were amplified by PCR from plasmid DNA templates: pNL1.1, pNL1.2 (Promega) and pCMV-Red Firefly Luc (Thermofisher) respectively. All oligonucleotide sequences are provided in S1 Table. Amplified genes were digested with *Bam*HI and *Kpn*I and ligated into pSSU-Neo [37] to produce the constructs pSSU-NanoLuc, pSSU-NanoLuc-PEST and pSSU-PRE9, for constitutive expression in *L*. *mexicana*. The pSSU expression vector contains flanking regions for integration into the rDNA locus of the parasite genome, and has been used in a number of *Leishmania* strains. [38–41] The three constructs (*Pac*I/*Mss*I digested) were transfected into mid-log *L*. *mexicana* procyclic promastigotes by nucleofection using a 4b Nucleofector system (Lonza), as described previously. [42] Transfectants were transferred to M199 agar plates containing 40 μg/ml Geneticin (Life Technologies) and clonal cell lines were established. Integration of the construct into the genome was assessed by PCR amplification of 50–100 ng total parasite DNA, using the oligonucleotide primers pSSU-F (region of the 18S gene) and pSSU-R (splice acceptor site in the pSSU vector). Total parasite DNA was purified from mid-log promastigote cells using the DNeasy Blood and Tissue Kit (Qiagen).

### Analysis of luciferase expression and stability

Parasite growth was counted at 24 hour intervals for 120 hours using a haemocytometer. Statistical significance was determined using a two way ANOVA, with the Dunnett Post-Hoc test for multiple comparisons (GraphPad Prism 6.0).

Detection of NanoLuc and NanoLuc-PEST enzymes was performed using the lytic Nano-Glo Assay (Promega), according to the manufacturer’s instructions. Detection of PRE9 was performed using either the Firefly Luciferase Glow Assay (Pierce), or the Bright-Glow Assay (Promega). Bioluminescence was measured using the Promega GloMax Multi Detection System. All data was normalised by subtracting bioluminescence values for the untransformed, parental line, and all assays done in triplicate.

Cycloheximide assays were performed over 8 hours. Cells were seeded to a cell density of 5 x10^5^ cells/well (100 μL/well). Cycloheximide (Sigma) was added to each well to a final concentration of 100 μM and incubated with cells for a range of time points between 0 and 8 hours. Bioluminescence was measured as described above, and the half-life of each luciferase was calculated by one phase decay non-linear regression (GraphPad Prism 6.0). Proteosome assays were performed using the method above with the addition of 5 μM MG-132 (Sigma) for 6 hours. [43] Statistical significance was determined using a paired, two-tailed T-test (GraphPad Prism 6.0).

### Macrophage assays

Toxicity assays were performed over 72 hours. THP-1 cells were differentiated into adherent macrophages, as described above. Cells were washed once in PBS to remove non-adherent monocytes, and compounds were added at varying dilutions. Cells were incubated with the compounds at 37°C with 5% CO_2_ for 72 hours. PrestoBlue (ThermoFisher) was added at a dilution of 1:10 per well. Plates were incubated in the dark at 37°C with 5% CO_2_ for 6 hours before reading on a Promega GloMax Multi Detection System (λ_ex_/λ_em_ = 525/580-640 nm).

For infection assays, the THP-1 cells were differentiated into adherent macrophages, as described above. Stationary phase *L*. *mexicana* metacyclic promastigotes were added at a ratio of 10:1 (parasites:macrophages) in complete RPMI media, and incubated at 32°C with 5% CO_2_ for 24 hours. Adherent macrophages were washed three times with PBS to remove extracellular parasites. Cells were then treated with 0.8 μM Amphotericin B (Fungizone, ThermoFisher) for 72 hours. Parasite load was determined using two methods; bioluminescence and indirect immunofluorescence. Bioluminescence was measured using the protocols described above. For indirect immunofluorescence, cells were fixed with 4% (v/v) formaldehyde (ThermoFisher) for 10 minutes. Cells were permeabilised with 0.1% (v/v) Triton-X 100 (Sigma) for 5 minutes, then blocked for 30 minutes with Image iT FX Signal Enhancer (Life Technologies). Cells were incubated with anti-HASPB [44] (1:250) for 1 hour, washed three times with PBS, then incubated with Alexa Fluor 594 conjugated goat-anti-rabbit IgG (Invitrogen) for 1 hour. Slides were washed three times with PBS, then mounted using Prolong Diamond Antifade Mountant (Life Technologies). Images were acquired using the EVOS FL Cell Imaging System (ThermoFisher), and parasite load assessed for a minimum of 100 macrophages per sample using the following equation:

$$Infection\;Index=\frac{Infected\;macrophages\;(\%)*Number\;of\;amastigotes}{Total\;number\;of\;macrophages}$$

### Amphotericin B testing and MMV Pathogen Box screening

The initial MMV Pathogen Box screen was performed on axenic amastigotes. Axenic amastigotes were seeded at a density of 1 x 10^5^/ml in duplicate (50 μl/well) in complete Schneider’s media pH 5.5. MMV Pathogen Box screening was performed using two concentrations of each compound: 2 and 10 μM. Hits were defined as compounds that, at a concentration of 2 μM, decreased the relative bioluminescence signal to 5% or less of that produced by the transgenic parasites incubated in solvent (DMSO) only. Both positive (Amphotericin B) and negative (equivalent volume of DMSO) controls were included on all plates. Assays were carried out as technical replicates with two independent assays performed (n = 4). Cells were incubated at 32°C with 5% CO_2_ for 72 hours, prior to viability measurements using either fluorescence or bioluminescence-based assays. For the fluorescence-based assays, AlamarBlue (ThermoFisher) was added at a dilution of 1:10 per well. Plates were incubated in the dark at 32°C with 5% CO_2_ for 6 hours before measuring the fluorescence on a Promega GloMax Multi Detection System (λ_ex_/λ_em_ = 525/580-640 nm). For bioluminescence, 20 μl of treated axenic amastigote culture was transferred in duplicate to a white 96-multiwell plate (Greiner, UK) and 20 μl of luciferase reagent (Nano-Glo Luciferase Assay buffer and Nano-Glo Luciferase Assay substrate, 200:1) was added to each well. After 3 minutes, the bioluminescence signal was measured using a Promega Glomax Multi Detection System. Results were analysed using GraphPad Prism 6.

For all assays, the percentage viability was calculated using the following equation:

$$Viability\;(\%)=100\;x\;\frac{\left[\mu (s)-\mu (-)\right]}{\left[\mu (+)-\mu (-)\right]}$$

Where μ(s) = mean value for the sample, μ(+) = mean of the DMSO only control, and μ(-) = mean of the positive control (2 μM Amphotericin B). For each screening assay, the assay quality parameters Zʹ score and signal:background ratios were calculated as described previously. [45]

## Results

### Luciferase activity and stability

**Fig 1:**
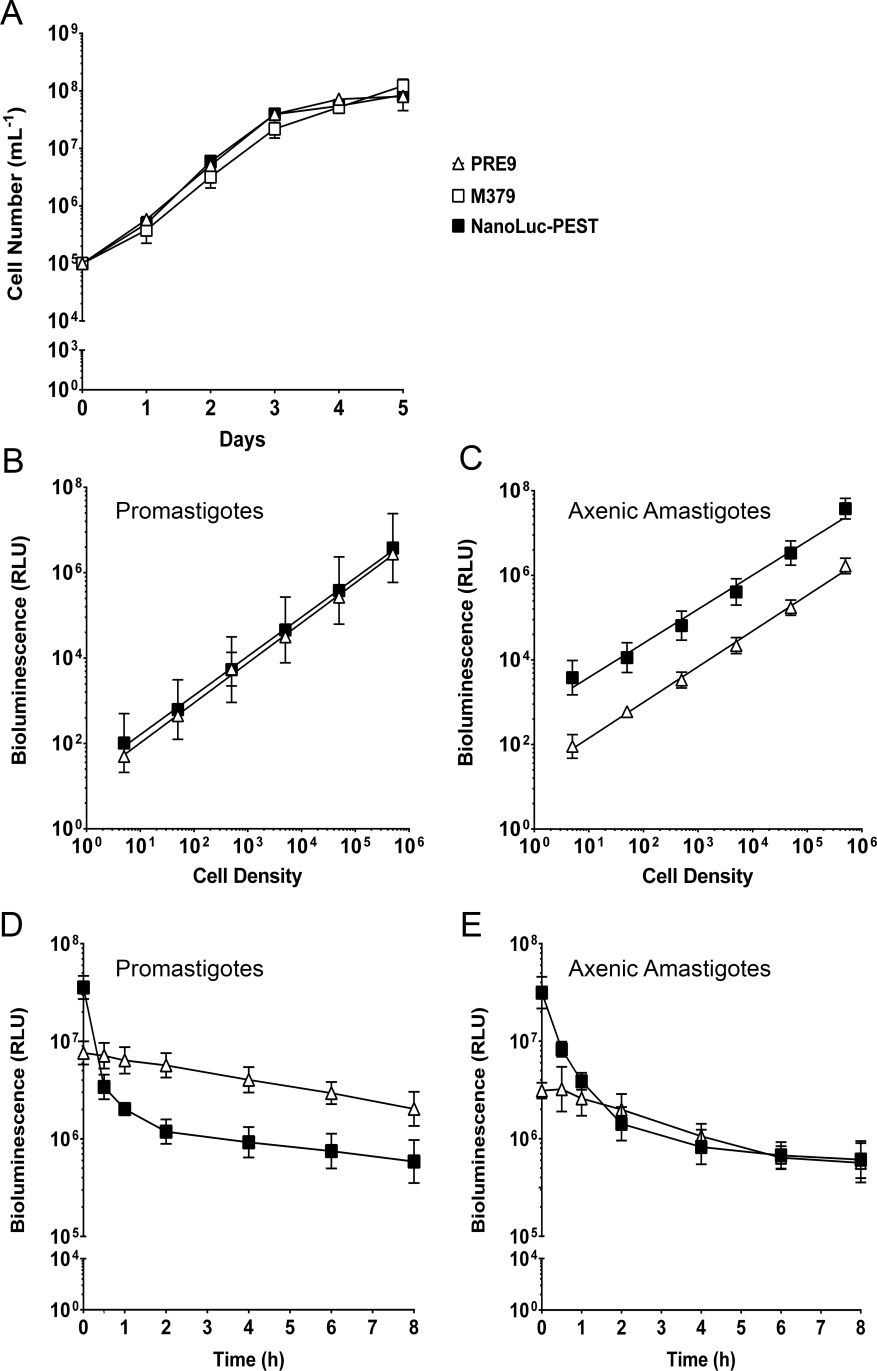
Characterisation of luciferase-expressing *L*. *mexicana* transgenic lines. (A) Promastigote growth curve of parental *L*. *mexicana* M379 (open square) and transgenic cell lines expressing PRE9 (open triangle) and NanoLuc-PEST (filled square) monitored over a five day time course. Mean values are shown (n = 3) ± SD. The Y-axis was transformed by log_10_. (B), (C) Cell density dilution series on the promastigote and axenic amastigote forms, respectively. Both X- and Y-axes were transformed by log_10_ prior to regression analysis. Mean values are shown (n = 3) ± SD. (D), (E) Cycloheximide assay on the promastigote and axenic amastigote forms, respectively, was monitored over an eight hour time course. The X-axis was transformed by log_10_ prior to regression analysis. Mean values are shown (n = 3) ± SD.

A panel of transgenic *L*. *mexicana* lines was generated that constitutively express either NanoLuc, NanoLuc-PEST or PRE9. Correct integration of the luciferase genes into the *L*. *mexicana* genome was assessed by PCR on total parasite DNA using primers complementary to the rRNA locus and the exogenous DNA (S1 Fig). A product of the expected size (900 bp), consistent with integration in the rRNA locus, was amplified from transgenic lines expressing NanoLuc-PEST and PRE9, but not from the NanoLuc cell line (S1A Fig). Amplification of the respective luciferase open reading frame was successful for all three lines (S1B Fig), suggesting that the introduced DNA in the NanoLuc line is episomal rather than integrated. The generation of an integrated NanoLuc cell line was not pursued as we found that the NanoLuc-PEST luciferase was a more dynamic indicator of cell viability (see below). Growth of the transgenic parasite lines was assessed by monitoring procyclic cells over a 5 day time period, and compared to the parental strain (Fig 1A, S2A Fig). There were comparable growth rates in all parasite lines over this period, showing no gross defects in fitness in the transgenic lines.

Expression of the luciferase enzymes was analysed by measuring bioluminescence in lysed cells. There was a significant linear correlation between cell number and bioluminescence over a wide range (5–500,000 cells per assay) in all luciferase-expressing cell lines, in both procyclic promastigotes and axenic amastigotes (Fig 1B and 1C respectively, S2B Fig). Luciferase activity (above background levels) was detected at less than 10 cells per well for all three luciferase enzymes, highlighting the sensitivity of the bioluminescence-based approach. Cells expressing NanoLuc showed considerably higher bioluminescence than those expressing the other two luciferases, with levels over 100-fold higher than the equivalent cells expressing PRE9 (S2 Fig). In comparison, the bioluminescence produced by NanoLuc-PEST was over 10 fold brighter than PRE9 in axenic amastigotes, whilst the signal intensity was comparable between the two luciferases in promastigotes (Fig 1B and 1C).

The half-life of each luciferase was determined by treatment with a supralethal dose of cycloheximide (100 μM), a known eukaryotic protein translation inhibitor. [46] Following an 8 hour incubation with cycloheximide, the bioluminescent signal from NanoLuc-expressing cells in both life cycle stages remained relatively constant, showing that this reporter is extremely stable (S2C Fig). In contrast, there was a 10-fold decrease in the bioluminescent signal after 1 hour in promastigotes and axenic amastigotes from the NanoLuc-PEST-expressing line (Fig 1D and 1E). DMSO, the solvent for cycloheximide, has no effect on bioluminescence levels at the volume used in this assay (S3 Fig). The calculated half-life for each of the luciferase enzymes was calculated to be >8 hours for NanoLuc (both parasite forms), 16 and 9 minutes for NanoLuc-PEST (axenic amastigotes and promastigotes, respectively) and 163 and 261 minutes for PRE9 (axenic amastigotes and promastigotes, respectively). Proteasome targeting of the NanoLuc-PEST enzyme was assessed in the presence of the proteasome inhibitor MG-132 (S4 Fig). [43] Addition of 5 μM MG-132 produced a statistically significant increase in bioluminescence relative to the DMSO control (S4A Fig; p = 0.0272 and 0.0007 in axenic amastigotes and promastigotes respectively). In the presence of both MG-132 and cycloheximide, bioluminescence values did decrease, but were still significantly higher than in the presence of cycloheximide alone (S4B Fig; p = 0.0219 and 0.0010 in axenic amastigotes and promastigotes respectively).

### Evaluation of luciferase-expressing *L*. *mexicana* cell lines for drug screening

In order to evaluate the NanoLuc-PEST enzyme as a dynamic reporter of anti-leishmanial activity, we compared this transgenic cell line against a standard resazurin-based fluorescent viability assay using Amphotericin B and Miltefosine (Table 1, S5 Fig). This was tested in axenic amastigotes. The robustness of the assays was assessed by calculating the Zʹ factor and signal:background (S:B) ratio. The EC_50_ values obtained from the parental and transgenic line using both bioluminescence- and fluorescence-based assays was similar (0.20–0.27 μM; Table 1, S5 Fig), and comparable to the EC_50_ value of 0.30 ± 0.02 μM previously described for *L*. *mexicana* axenic amastigotes against Amphotericin B. [47] All assays had a calculated Zʹ factor value of ≥ 0.64, demonstrating the robustness of each assay (defined as a Zʹ value greater than 0.5). [48] However, there were marked differences in the S:B ratio. The bioluminescence-based assay displayed a S:B ratio between 50- and 100-fold higher than the standard fluorescence-based assay on the same cell line (Table 1).

**Table 1:** EC_50_ values and assay parameters following treatment with Amphotericin B and Miltefosine against wild-type and NanoLuc-PEST expressing *L*. *mexicana* (see S5 Fig for full dataset).

Strain	Drug	Assay	EC_50_ (μM)	95% CI	Zʹ	Signal:Background
***Parental***	Amphotericin B	Fluorescence	0.23	0.23–0.29	0.64–0.88	3.2–3.6
	Miltefosine		1.11	0.99–1.16	0.68–0.72	2.2–3.3
***NanoLuc-PEST***	Amphotericin B	Fluorescence	0.27	0.27–0.28	0.72–0.88	2.4–5.1
	Miltefosine		1.99	1.8–2.06	0.70–0.83	4.2–4.5
	Amphotericin B	Bioluminescence	0.20	0.18–0.20	0.79–0.90	322.5–369.8
	Miltefosine		2.19	2.07–2.35	0.84–0.86	192.7–211.5

**Fig 2:**
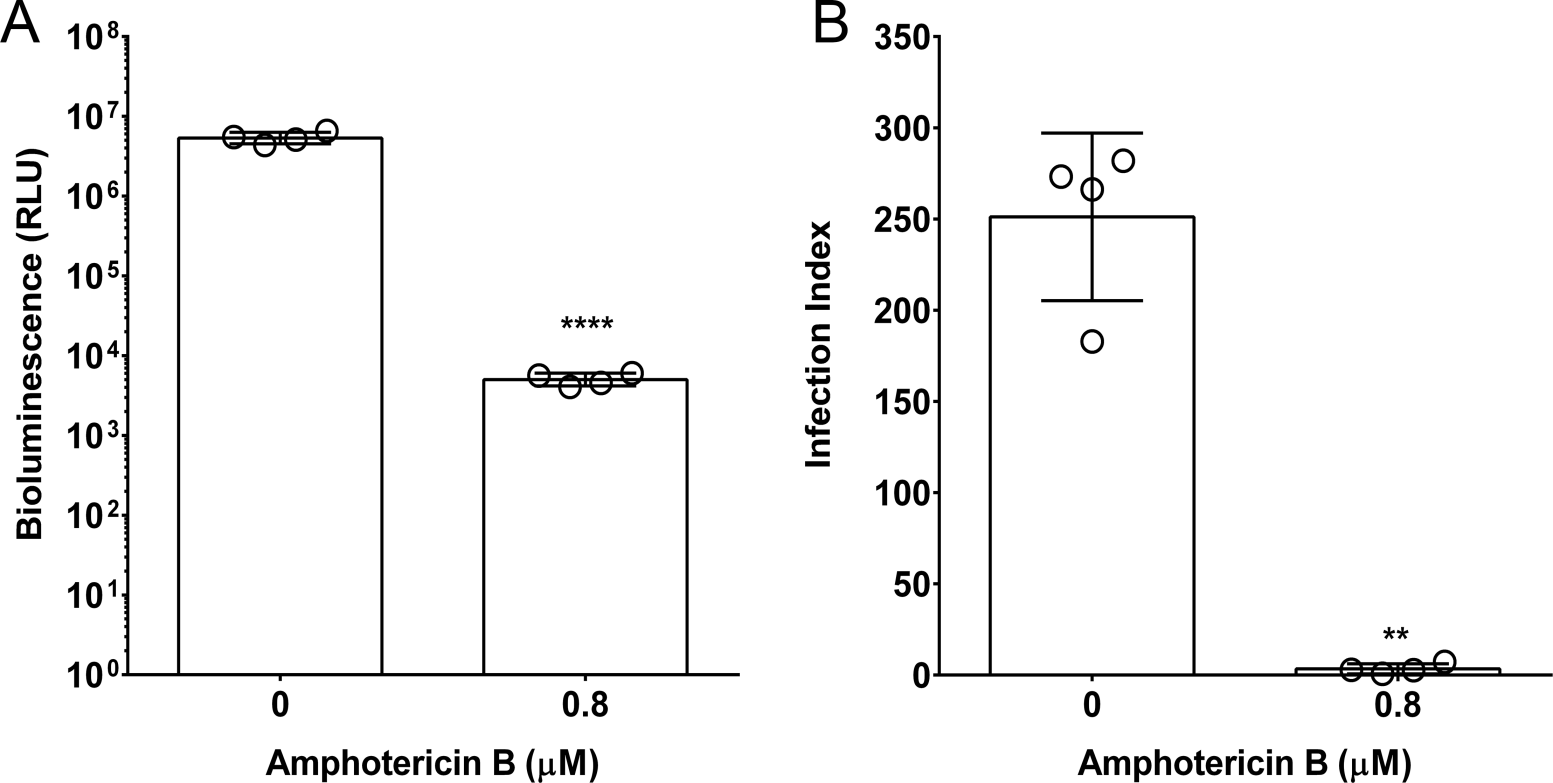
Comparison of bioluminescence- and microscopy-based intramacrophage infection assays following treatment with a supralethal dose of Amphotericin B. (A) The bioluminescence-based assay was used to assess infection of PMA-differentiated THP-1 macrophages by stationary phase *L*. *mexicana* NanoLuc-PEST promastigotes. Infected cells were exposed to 0.8 μM Amphotericin B, or left untreated, for 72 hours. Mean values are shown (n = 4) ± SD. The Y-axis was transformed by log_10_, and the data was analysed by paired, two-tailed T-test (*p*<0.001). (B) The standard microscopy-based counting assay was used to assess infection of PMA-differentiated THP-1 macrophages by stationary phase *L*. *mexicana* NanoLuc-PEST promastigotes Infected cells were exposed to 0.8 μM Amphotericin B, or left untreated, for 72 hours. Infection indices were calculated as described in the methods. Mean values are shown (n = 4) ± SD. The data was analysed by paired, two-tailed T-test on the infection index data (*p* = 0.0017).

We then evaluated the potential of the NanoLuc-PEST cell line to determine parasite viability in an intramacrophage assay, and compared this to the standard microscopy-based counting assay. We observed a correlation between the bioluminescence- and microscopy-based methods (Fig 2); specifically, the infection index and the bioluminescent signal decreased in the presence of a supralethal dose of Amphotericin B by ≥99% (Fig 2).

### MMV Pathogen Box screening

**Fig 3:**
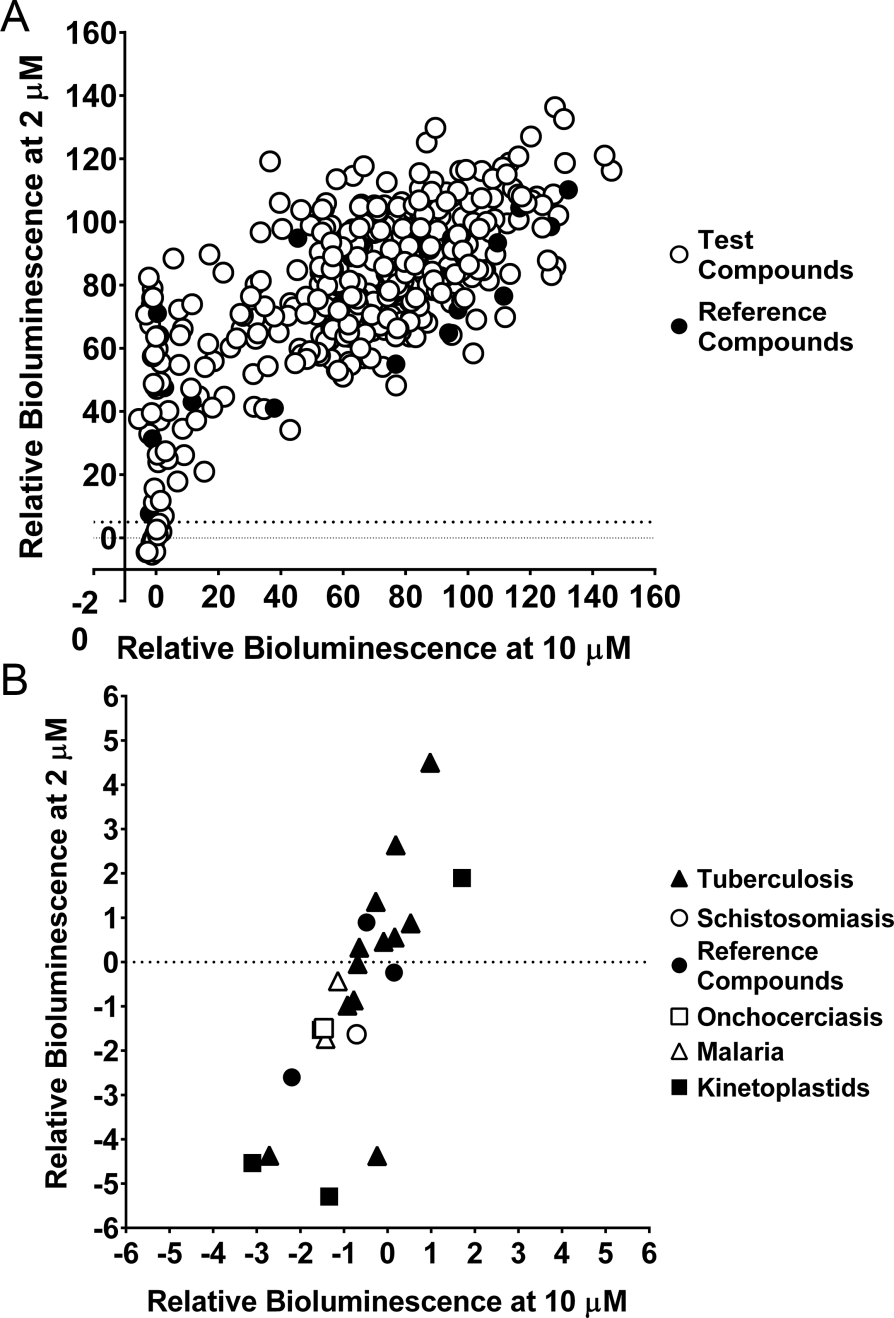
Screening the MMV Pathogen Box against NanoLuc-PEST expressing axenic amastigotes, using bioluminescence. (A) Relative bioluminescence (compared to untreated controls) of *L*. *mexicana* expressing NanoLuc-PEST was measured following treatment with compounds at two concentrations (2 and 10 μM). Reference compounds in the MMV Pathogen Box (see S2 Table) are shown as filled circles. Mean values are shown (n = 4). The dotted line indicates the level of relative bioluminescence (5% or less at 2 μM) that forms our cut off for compounds to be considered hits. (B) Represents the bottom left of Fig 3A, and shows the 23 compounds identified as hits from our screen of the MMV Pathogen Box. These compounds are depicted as the MMV Pathogen Box disease set: tuberculosis (filled triangle), schistosomiasis (open circle), onchocerciasis (open square), malaria (open triangle), kinetoplastids (filled square) and reference compounds (filled circle). The reference compounds shown here are Buparvaquone, Mebendazole and Auranofin. Mean values are shown (n = 4) from two independent experiments.

Our findings indicate that the NanoLuc-PEST transgenic cell line is the most dynamic reporter of those evaluated here. In addition, it is sensitive and robust in both the axenic and intramacrophage assay formats. Consequently, we evaluated this system for compound screening. We used a two-step process to do this: i) identify hits against axenic amastigotes, ii) screen these hits using our bioluminescence-based intramacrophage assay. Prior to this, the assay was optimised to reduce the volume and cell concentration while maintaining a linear readout in a 96-well microplate format (S6 Fig). The MMV Pathogen Box resource, comprising 400 diverse drug-like molecules, was tested at two concentrations (10 μM and 2 μM) on axenic amastigotes (S7 Fig), and are summarised in Fig 3. Hits were defined as compounds that, at a concentration of 2 μM, decreased the relative bioluminescence signal to 5% or less of that produced by the transgenic parasites incubated in solvent (DMSO) only (Fig 3B). The complete dataset is depicted graphically in S7 Fig, and in S2 Table. A total of 23 hits were identified (Fig 3B), 3 of which were the reference compounds Buparvaquone, Mebendazole and Auranofin. Of these 23 identified hits, 52% originated from *Mycobacterium tuberculosis* screening programmes.

**Fig 4:**
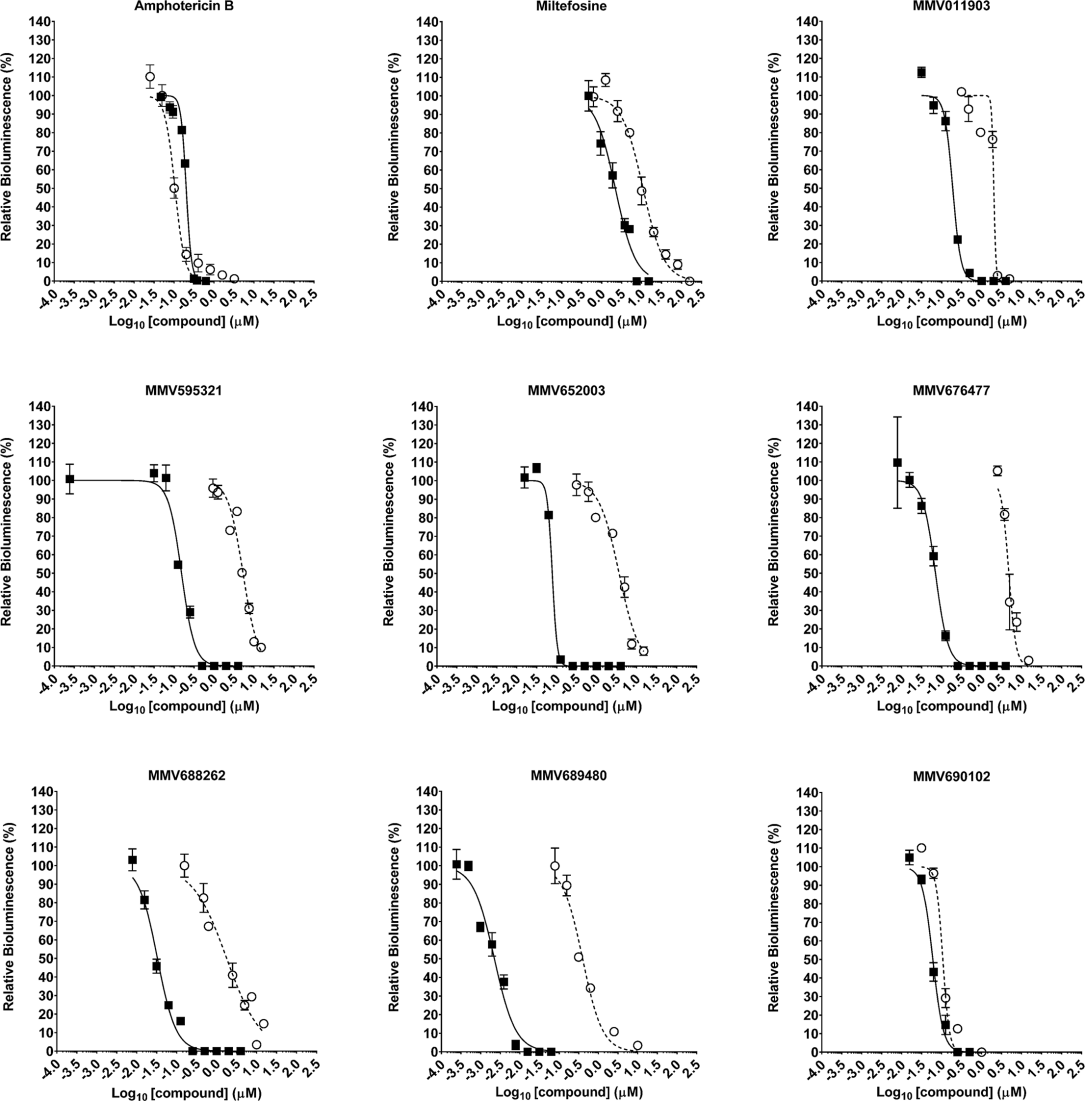
EC_50_ responses to seven MMV Pathogen Box compounds in both axenic and intramacrophage assays, compared to Amphotericin B and Miltefosine. The response of *L*. *mexicana* expressing NanoLuc-PEST when screened against seven hit compounds, and the two controls Amphotericin B and Miltefosine. Parasites were screened as either axenic amastigotes (filled square) or as intramacrophage amastigotes (open circle) in the THP-1 infection model. Mean values are shown (n = 4) ± SD from two independent experiments. EC_50_ and EC_90_ values are detailed in Table 2.

All 23 hits were analysed to determine their EC_50_ (S3 Table), except for Mebendazole (MMV003152), which could not be resolved. Eight of these compounds displayed an EC_50_ value less that that observed for Amphotericin B (0.2 μM). Of these 8 compounds, 2 were reference compounds (Buparaquone and Auranofin). We picked the most potent reference compound (Buparaquone) and the 6 test compounds to screen using the intramacrophage assay in parallel with Amphotericin B and Miltefosine (Table 2, Fig 4). There was an increase in the EC_50_ values from the intramacrophage assay for all 7 compounds when compared to the EC_50_ values from the axenic amastigote screen (Table 2, S3 Table and Fig 4). EC_90_ values from the intramacrophage assay are also included (Table 2). Of these 7 compounds, 5 are known to be active against kinetoplastids, with 4 of these active against *Leishmania spp* (Table 2). Our results for MMV690102 correlate well with the existing data. [49–51] However, our EC_50_ results for MMV689480 (Buparvaquone) in the intramacrophage assay is at least three times lower than the previously reported values against *L*. *mexicana*. [52] Our results for MMV688262 (Delamanid) and MMV595321 were higher than previously reported values against *L*. *donovani* (Table 2) [49, 53, 54].

**Table 2:** Activity of the six most potent MMV compounds against axenic and intramacrophage amastigotes. Previous EC_50_ data for compounds against *Leishmania spp*. are shown in italics, where available.

Compound	EC_50_ (μM) against amastigotes		EC_90_ (μM) against amastigotes		MMV Disease Set	Known Activity Against *Leishmania spp*.?
	Axenic	Intramacrophage	Axenic	Intramacrophage		
Amphotericin B	0.201 (*0*.*303*)	0.105 *(0*.*271/0*.*009)*	0.279	0.185	NA	Yes [47, 55]
Miltefosine	1.99	10.87 (*15*.*7*)	19.76	37.27	NA	Yes [55]
MMV676477	0.069	4.783	0.168	6.107	Tuberculosis	No
MMV652003	0.077	3.637	0.104	10.670	Kinetoplastid	No–but the compound class is active against *T*. *brucei* [56–58]
MMV011903	0.189	2.015	0.320	2.286	Malaria	No
MMV689480	0.002	0.394 *(1*.*25/1*.*44)*	0.011	1.260	Reference Compound (Buparvaquone)	Yes [52]
MMV595321	0.153 (*0*.*501*)	5.295 *(3*.*162)*	0.357	11.170	Kinetoplastid	Yes [49, 53]
MMV690102	0.060 (*0*.*040*)	0.107 *(0*.*100)*	0.123	0.160	Kinetoplastid	Yes [49–51]
MMV688262	0.033 *(0*.*005)*	1.780 *(0*.*0865–0*.*298)*	0.112	25.070	Tuberculosis (Delamanid)	Yes [54]

Compounds supplied in the MMV Pathogen Box have been tested for cytotoxicity against human cell lines, and this information is available online (https://www.pathogenbox.org/about-pathogen-box/supporting-information). We have summarised this existing data alongside results from cytotoxicity screens against the THP-1 cell line for the compounds that we had sufficient available material (S4 Table). Tested compounds displayed an EC_50_ > 50 μM.

## Discussion

A key issue with the anti-leishmanial drug discovery process is the step from axenic amastigotes to *in vivo* models. The compromise–using an *in vitro* intramacrophage assay–involves the use of laborious and time consuming microscopy-based techniques that assess parasite burden, but cannot determine parasite viability. This paper describes the use of a tractable bioluminescent marker (NanoLuc-PEST) that correlates specifically with parasite viability. This method uses a simple and robust bioluminescence assay that decreases the time required to screen compounds against intramacrophage amastigotes *in vitro*, and provides an environment that should be more similar to the *in vivo* models.

All three of the luciferases tested enabled the detection of less than 10 cells per well (Fig 1B and 1C, S2 Fig), demonstrating the highly sensitive nature of the bioluminescence approach. Luciferase activity was not significantly greater in axenic amastigotes relative to promastigotes for NanoLuc and NanoLuc-PEST lines, although the inserted genes were fused to the CPS intergenic region, which has previously resulted in the upregulation of GFP in the amastigote stage. [59] However, the NanoLuc and NanoLuc-PEST luciferases displayed higher bioluminescence signals in the axenic amastigote form when compared to the PRE9 alternative (Fig 1C). There are several factors that may be involved in this. Specifically, the NanoLuc luciferase was the only one of the three tested that was not genomically integrated (S1A Fig), therefore it may have higher copy numbers compared to the NanoLuc-PEST and PRE9 enzymes. Differences in bioluminescence may also stem from the use of different substrates, specifically furimazine (for the NanoLuc and PEST derivative) and beetle luciferin (for PRE9). However, it is the sensitivity and brightness produced by the NanoLuc enzyme, and its PEST variant, that makes them highly attractive for screening purposes.

Signal intensity is not the only factor that should be taken into consideration when assessing a reporter molecule. The signal must also correlate with cell viability. The NanoLuc version of the enzyme is very stable, with a half-life greater than 8 hours (S2 Fig). The addition of the PEST domain, which is comprised of a proline, glutamic acid, serine and threonine rich sequence, [60] targets the enzyme for degradation by the 26S proteasome (S4 Fig). [61] This makes the NanoLuc-PEST variant a more dynamic reporter for cell viability. Whilst the Zʹ values for both the bioluminescence and fluorescence based assays were above 0.5 (the lower limit for an acceptable screening assay [48]), the S:B ratios for the NanoLuc-PEST transgenic cell line was 50- to 100-fold higher than the standard fluorescence-based assay (Table 1). The higher S:B ratio of NanoLuc-PEST reflects the relatively high enzymatic activity and short half-life of this protein, contributing to the greater dynamic range achieved using this modified reporter. Consequently, it was the clonal, genomically integrated NanoLuc-PEST cell line that we felt was most appropriate for further testing.

The main advantage with this bioluminescent technique is its potential for use with intramacrophage assays. The current gold standards for intramacrophage compound screening are microscopy-based assays. The microscopy techniques use nuclear staining, parasite-specific antibodies or stable reporter molecules. [11–15, 36, 62] These rely on the use of fluorescence, which can be affected by potential autofluorescence of test compounds. Furthermore, these methods rely solely on detecting the presence of the parasite, not determining parasite viability. We directly compared the potential of the NanoLuc-PEST cell line using the bioluminescence- and counting-based assays on paired samples. Our results from the bioluminescence-based assay mirrored that of the microscopy-based assay (Fig 2); specifically the level of infection decreased by ≥99%. Our bioluminescent intramacrophage assay is not only more sensitive, but has the potential to be automated, and would therefore allow large-scale screening of compound libraries using a more relevant *in vitro* assay. The NanoLuc-PEST expressing cell line thus provides a useful tool to assess compound efficacy against intracellular parasites without the need for arduous, less sensitive, microscopy-based assays. Expressing the NanoLuc-PEST protein in other *Leishmania* species for screening purposes is also possible, as the pSSU-Int expression vector has been used successfully in *L*. *donovanni*, [39] *L*. *major*, [37, 40] and *L*. *infantum* [37, 38, 41].

One limitation of the bioluminescent assay is that it requires cell lysis. This means that the cells, once measured, cannot then be used in additional, downstream applications. However, the volumes required for lysis is small (<100 μL per technical replicate), so an aliquot can be taken and tested from a larger sample, leaving the remaining material for further analysis. A second limitation is that any extracellular promastigotes present within the well will produce a signal when lysed. Whilst every care was taken to ensure the macrophages were washed carefully, we cannot guarantee that all of the extracellular promastigotes were removed. However, incubation at 32°C should induce differentiation into axenic amastigotes, which are less likely to be retained on the macrophage surface during subsequent wash steps.

We then tested the NanoLuc-PEST cell line against the MMV Pathogen Box. The EC_50_ values obtained from the intramacrophage assay were higher than those values obtained from the screen of the axenic amastigotes alone (Table 2). This is perhaps expected, as the compound must traverse two additional membranes before it reaches the *Leishmania* amastigote. However, all seven compounds displayed an EC_50_ < 6 μM, which is below the 10 μM limit detailed for hits against intracellular *L*. *donovani*. [63] Of these seven compounds, five are known to be active against kinetoplastids, with four active against *Leishmania spp* (Table 2). Our results for MMV690102 correlate well with the existing data, despite this previous data being gathered against *L*. *donovani*. However, our EC_50_ results for MMV689480 (Buparvaquone) in the intramacrophage assay is at least three times lower than the previously reported values against *L*. *mexicana*. This may indicate the increased sensitivity of our intramacrophage assay, as only viable parasites within the macrophage are detected. Our results for MMV688262 (Delamanid) and MMV595321 were higher than previously reported values (Table 2), which may be due to species variation.

In conclusion, we have demonstrated the use of transgenic *L*. *mexicana* expressing a novel luciferase as a tractable, rapid and sensitive system for compound screening, using the MMV Pathogen Box as proof of principle. This system has the added advantage of allowing detection of parasite viability in an *in vitro* infected macrophage model, a method that has previously been shown to be advantageous for studying *Plasmodium falciparum* and *Mycobacterium tuberculosis* in an intracellular environment. [16, 17] This allows screening programmes to assess compound activity against the intracellular parasite without the time burden, requirement for specialist equipment and post-assay processing associated with the current, microscopy-based, techniques.

## Supporting information

S1 Fig Molecular analysis of luciferase-expressing transgenic *L. mexicana* cell lines. (A) Integration of the luciferase constructs into the rDNA locus was assessed by PCR amplification from total parasite DNA using the oligonucleotides pSSU-F and pSSU-R (S1 Table). (B) Presence of the specific luciferase genes in the total parasite DNA was assessed by PCR using the appropriate cloning oligonucleotides (S1 Table). WT; parental, NL; NanoLuc, NP; NanoLuc-PEST, PR; red-shifted firefly luciferase (PRE9).
(TIF)

S2 Fig Characterisation of the episomal NanoLuc luciferase expressed in *L. mexicana*. (A) Promastigote growth curve of the parental *L*. *mexicana* M379 (open square) and the cell line expressing NanoLuc (closed circle). Mean values are shown (n = 3) ± SD. The Y-axis was transformed by log_10_. (B) Cell density dilution series on the promastigote (filled circle) and axenic amastigote (open circle) forms. Both X- and Y-axes were transformed by log_10_ prior to regression analysis. Mean values are shown (n = 3) ± SD. (C) Cycloheximide assay on the promastigote (filled circle) and axenic amastigote (open circle) forms was monitored over an eight hour time course. The X-axis was transformed by log_10_ prior to regression analysis. Mean values are shown (n = 3) ± SD.
(TIF)

S3 Fig Effect of DMSO on the cycloheximide assay system. (A) DMSO (volume equivalent to 100 μM cycloheximide) was incubated for eight hours with transgenic promastigote forms expressing NanoLuc (filled circle), Rluc (open triangle) and NanoLuc-PEST (filled square), and compared to untreated controls. Mean values are shown (n = 3) ± SD. The Y-axis was transformed by log_10_. (B) DMSO (volume equivalent to 100 μM cycloheximide) was incubated for eight hours with transgenic axenic amastigote forms expressing NanoLuc (filled circle), Rluc (open triangle) and NanoLuc-PEST (filled square), and compared to untreated controls. Mean values are shown (n = 3) ± SD. The Y-axis was transformed by log_10_.
(TIF)

S4 Fig Proteasome targeting of the NanoLuc-PEST enzyme. (A) Response of the assay system to the proteasome inhibitor MG-132 compared to the DMSO control. Mean values are shown (n = 3) ± SD. The Y-axis was transformed by log_10_, and the data was analysed by paired, two-tailed T-test on normalised data (*p* = 0.0272 and 0.0007 for axenic amastigotes and promastigotes respectively). (B) Response of the assay system to the protein synthesis inhibitor cycloheximide in the presence and absence of the proteasome inhibitor MG-132. Mean values are shown (n = 3) ± SD. The Y-axis was transformed by log_10_, and the data was analysed by paired, two-tailed T-test on normalised data (*p* = 0.0219 and 0.0010 for axenic amastigotes and promastigotes respectively).
(TIF)

S5 Fig Amphotericin B and Miltefosine concentration dependent response against parental, NanoLuc and NanoLuc-PEST axenic amastigotes. (A) Response of the parental M379 *L*. *mexicana* cell line to Amphotericin B (filled circles) and Miltefosine (filled squares), measured using the fluorescence-based AlamarBlue assay. Mean values are shown (n = 6) ± SD. (B) Response of the transgenic NanoLuc expressing *L*. *mexicana* cell line to Amphotericin B, measured using both the fluorescence-based AlamarBlue assay (filled circles) and the bioluminescence-based assay (open circles). Mean values are shown (n = 6) ± SD. (C) Response of the transgenic NanoLuc-PEST expressing *L*. *mexicana* cell line to Amphotericin B, measured using both the fluorescence-based AlamarBlue assay (filled circles) and the bioluminescence-based assay (open circles). Mean values are shown (n = 6) ± SD. (D) Response of the transgenic NanoLuc-PEST expressing *L*. *mexicana* cell line to Miltefosine, measured using both the fluorescence-based AlamarBlue assay (filled squares) and the bioluminescence-based assay (open squares). EC_50_ values for the parental and NanoLuc-PEST cell lines are reported in Table 1.
(TIF)

S6 Fig Miniaturising the Nano-Glo assay. (A) *L*. *mexicana* axenic amastigotes (1 x 10^5^ cells/ml) expressing NanoLuc-PEST were treated with the EC_50_ dose of Amphotericin B (0.2 μM). Following a 72 hour incubation, different volumes were taken and added to a fixed volume of 50 μL of the Nano-Glo reagent (lysis buffer and substrate, diluted 200:1), and complete Schneider’s medium pH 5.5 added to a final volume of 100 μL. The data shows a linear response down to a cell volume of 10 μL. We chose a final cell volume of 20 μL for further screening assays. Mean values are shown of three technical replicates ± SD. (B) Axenic amastigotes (1 x 10^5^ cells/ml) expressing NanoLuc-PEST were treated with the EC_50_ dose of Amphotericin B (0.2 μM). Using the 20 μL cell volume determined in (A), the axenic amastigotes were exposed to different volumes of the Nano-Glo reagent (up to 2.5x the cell volume). No difference in the bioluminescence values in any of the assay conditions was observed. A total assay volume of 40 μL, comprised of 20 μL axenic amastigotes and 20 μL Nano-Glo reagent was then selected for the MMV Pathogen Box Screen. Mean values are shown of three technical replicates ± SD.
(TIF)

S7 Fig Graphical representation of the complete MMV Pathogen Box screen against *L. mexicana* NanoLuc-PEST axenic amastigotes. The relative bioluminescence (%) of the *L*. *mexicana* expressing NanoLuc-PEST when screened against two compound concentrations: 2 μM (filled circle) and 10 μM (open circle). The MMV Pathogen Box contains five plates, with 80 compounds per plate. The data for each plate is provided as a graph labelled with the plate identifier. (A) Plate A. (B) Plate B. (C) Plate C. (D) Plate D. (E) Plate E. Dashed lines indicate a decrease in bioluminescence of 5%. Mean values are shown (n = 4) ± SD from two independent experiments. Also see S2 Table.
(TIF)

S1 Table Oligonucleotide sequences for cloning and integration. (DOCX)

S2 Table Relative bioluminescence (%) following the MMV Pathogen Box screen at two compound concentrations (2 μM and 10 μM), against axenic amastigotes expressing NanoLuc-PEST. Red values indicate the most potent compounds at 2 μM, blue values indicate the least potent compounds at 2 μM.
(DOCX)

S3 Table EC_50_ values for ‘hit’ compounds, from the axenic amastigote screen. EC_50_ values are colour-coded, where red indicates the most potent compounds, and blue indicates the least potent compounds.
(DOCX)

S4 Table Cytotoxicity data for ‘hit’ compounds. Data is obtained either from the MMV Pathogen Box website ^(a)^, or experimentally ^(b)^.
(DOCX)
