# Supplementary figures and images for: Development of NanoLuc-PEST expressing Leishmania mexicana as a new drug discovery tool for axenic- and intramacrophage-based assays
Source: PLoS Negl Trop Dis. 2018 Jul 12;12(7):e0006639. doi: 10.1371/journal.pntd.0006639 (PMC6057649; doi:10.1371/journal.pntd.0006639)

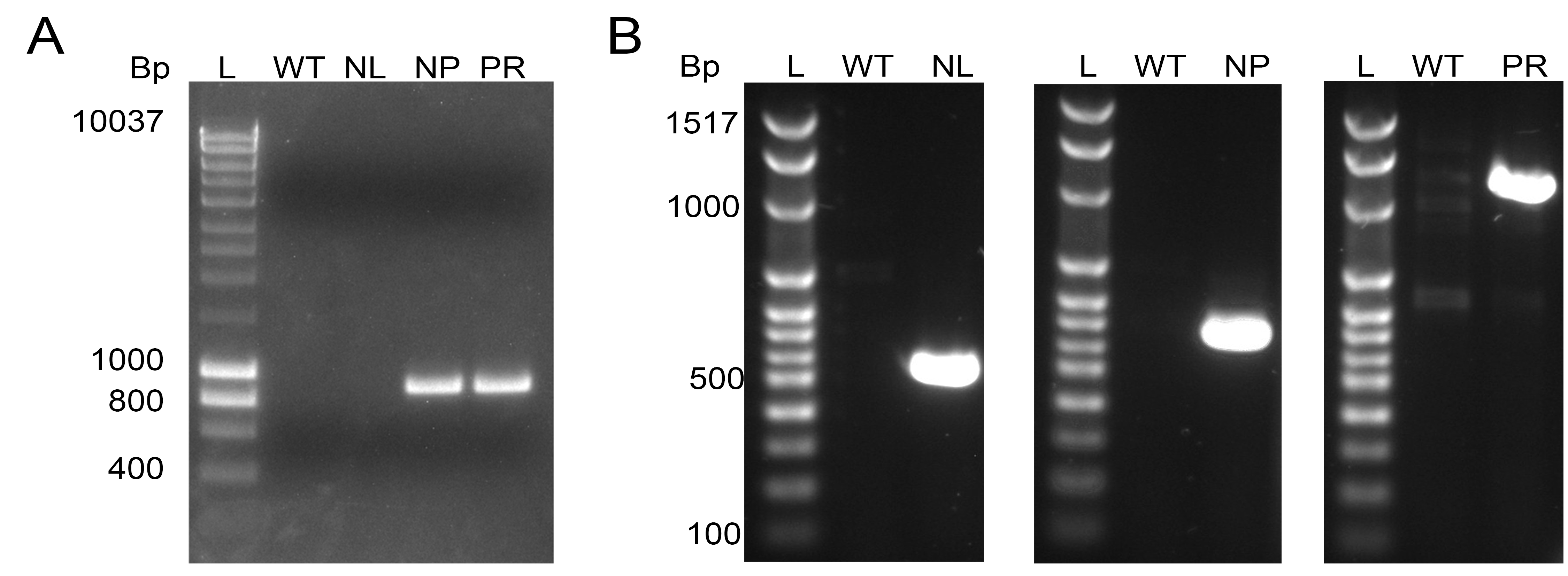

Supplement: S1 Fig — (A) Integration of the luciferase constructs into the rDNA locus was assessed by PCR amplification from total parasite DNA using the oligonucleotides pSSU-F and pSSU-R (S1 Table). (B) Presence of the specific luciferase genes in the total parasite DNA was assessed by PCR using the appropriate cloning oligonucleotides (S1 Table). WT; parental, NL; NanoLuc, NP; NanoLuc-PEST, PR; red-shifted firefly luciferase (PRE9). (TIF) [file pntd.0006639.s001.tif]

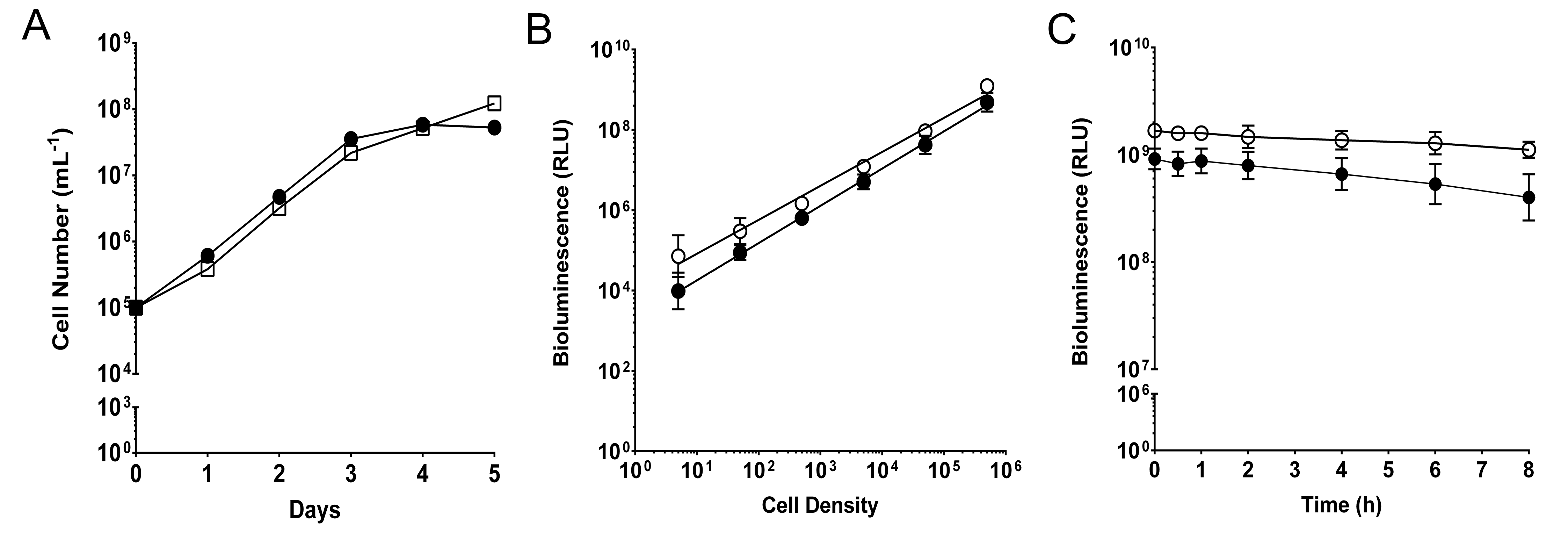

Supplement: S2 Fig — (A) Promastigote growth curve of the parental L. mexicana M379 (open square) and the cell line expressing NanoLuc (closed circle). Mean values are shown (n = 3) ± SD. The Y-axis was transformed by log10. (B) Cell density dilution series on the promastigote (filled circle) and axenic amastigote (open circle) forms. Both X- and Y-axes were transformed by log10 prior to regression analysis. Mean values are shown (n = 3) ± SD. (C) Cycloheximide assay on the promastigote (filled circle) and axenic amastigote (open circle) forms was monitored over an eight hour time course. The X-axis was transformed by log10 prior to regression analysis. Mean values are shown (n = 3) ± SD. (TIF) [file pntd.0006639.s002.tif]

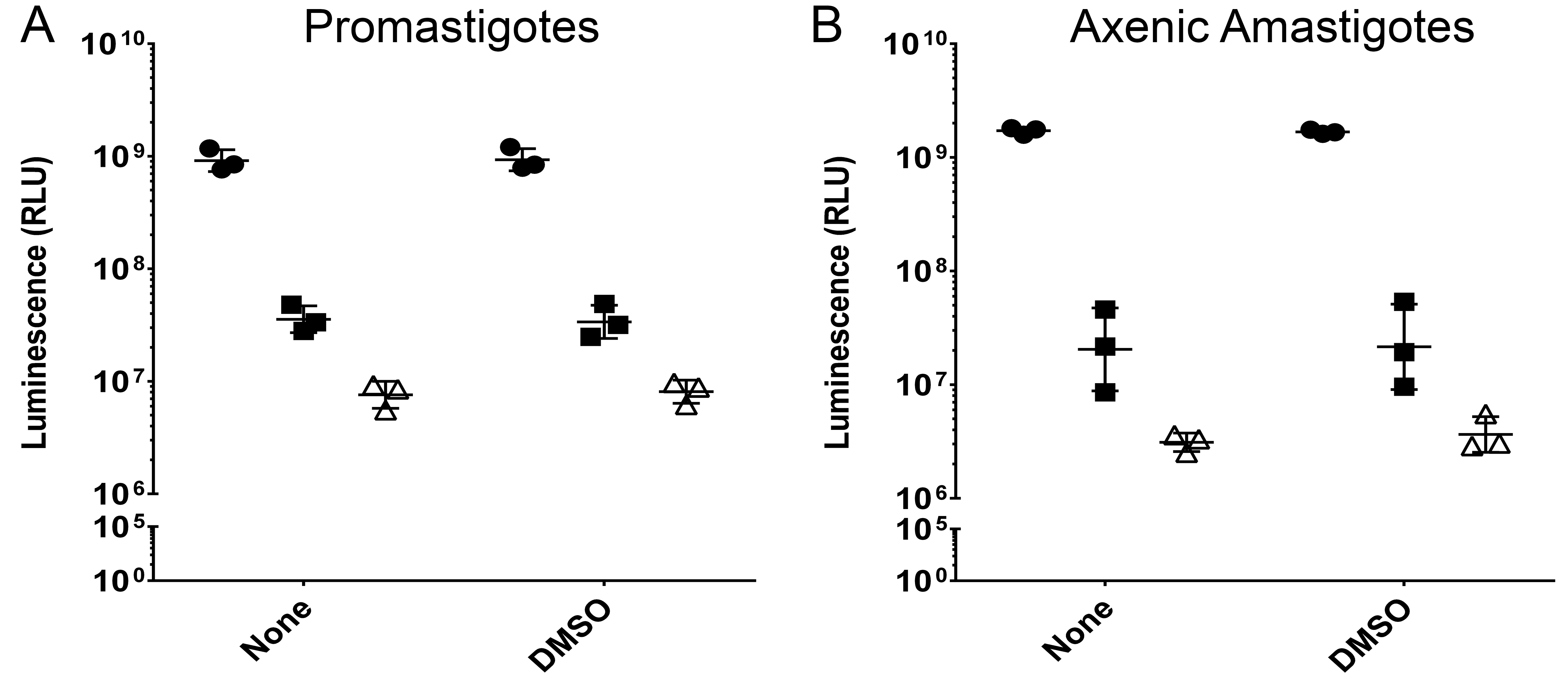

Supplement: S3 Fig — (A) DMSO (volume equivalent to 100 μM cycloheximide) was incubated for eight hours with transgenic promastigote forms expressing NanoLuc (filled circle), Rluc (open triangle) and NanoLuc-PEST (filled square), and compared to untreated controls. Mean values are shown (n = 3) ± SD. The Y-axis was transformed by log10. (B) DMSO (volume equivalent to 100 μM cycloheximide) was incubated for eight hours with transgenic axenic amastigote forms expressing NanoLuc (filled circle), Rluc (open triangle) and NanoLuc-PEST (filled square), and compared to untreated controls. Mean values are shown (n = 3) ± SD. The Y-axis was transformed by log10. (TIF) [file pntd.0006639.s003.tif]

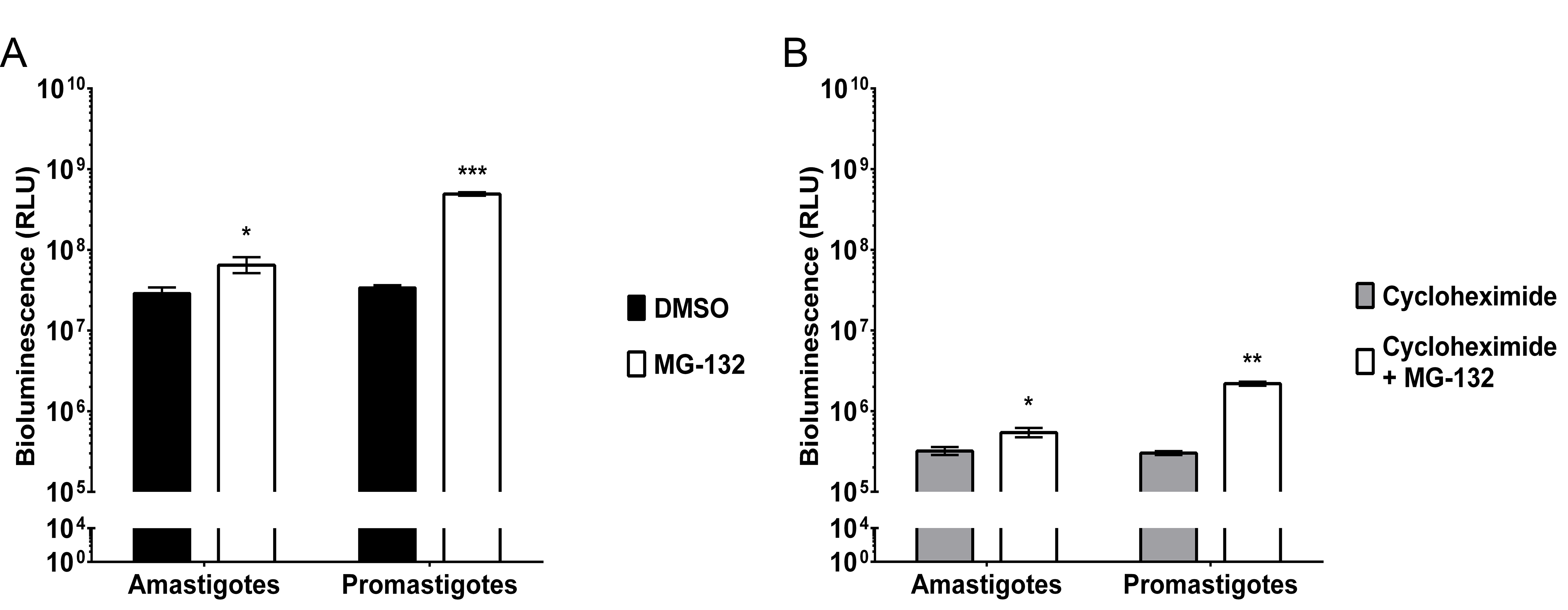

Supplement: S4 Fig — (A) Response of the assay system to the proteasome inhibitor MG-132 compared to the DMSO control. Mean values are shown (n = 3) ± SD. The Y-axis was transformed by log10, and the data was analysed by paired, two-tailed T-test on normalised data (p = 0.0272 and 0.0007 for axenic amastigotes and promastigotes respectively). (B) Response of the assay system to the protein synthesis inhibitor cycloheximide in the presence and absence of the proteasome inhibitor MG-132. Mean values are shown (n = 3) ± SD. The Y-axis was transformed by log10, and the data was analysed by paired, two-tailed T-test on normalised data (p = 0.0219 and 0.0010 for axenic amastigotes and promastigotes respectively). (TIF) [file pntd.0006639.s004.tif]

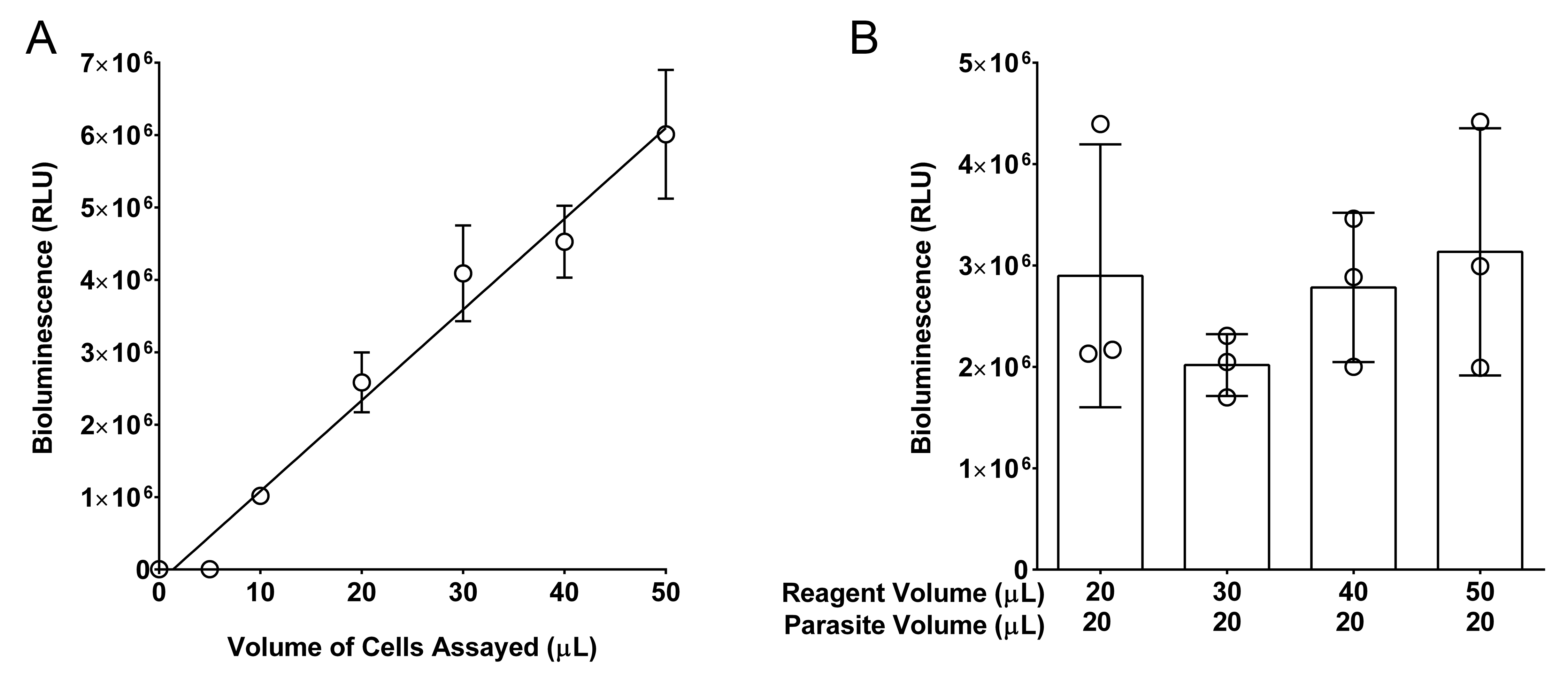

Supplement: S6 Fig — (A) L. mexicana axenic amastigotes (1 x 10^5^ cells/ml) expressing NanoLuc-PEST were treated with the EC50 dose of Amphotericin B (0.2 μM). Following a 72 hour incubation, different volumes were taken and added to a fixed volume of 50 μL of the Nano-Glo reagent (lysis buffer and substrate, diluted 200:1), and complete Schneider’s medium pH 5.5 added to a final volume of 100 μL. The data shows a linear response down to a cell volume of 10 μL. We chose a final cell volume of 20 μL for further screening assays. Mean values are shown of three technical replicates ± SD. (B) Axenic amastigotes (1 x 10^5^ cells/ml) expressing NanoLuc-PEST were treated with the EC50 dose of Amphotericin B (0.2 μM). Using the 20 μL cell volume determined in (A), the axenic amastigotes were exposed to different volumes of the Nano-Glo reagent (up to 2.5x the cell volume). No difference in the bioluminescence values in any of the assay conditions was observed. A total assay volume of 40 μL, comprised of 20 μL axenic amastigotes and 20 μL Nano-Glo reagent was then selected for the MMV Pathogen Box Screen. Mean values are shown of three technical replicates ± SD. (TIF) [file pntd.0006639.s006.tif]

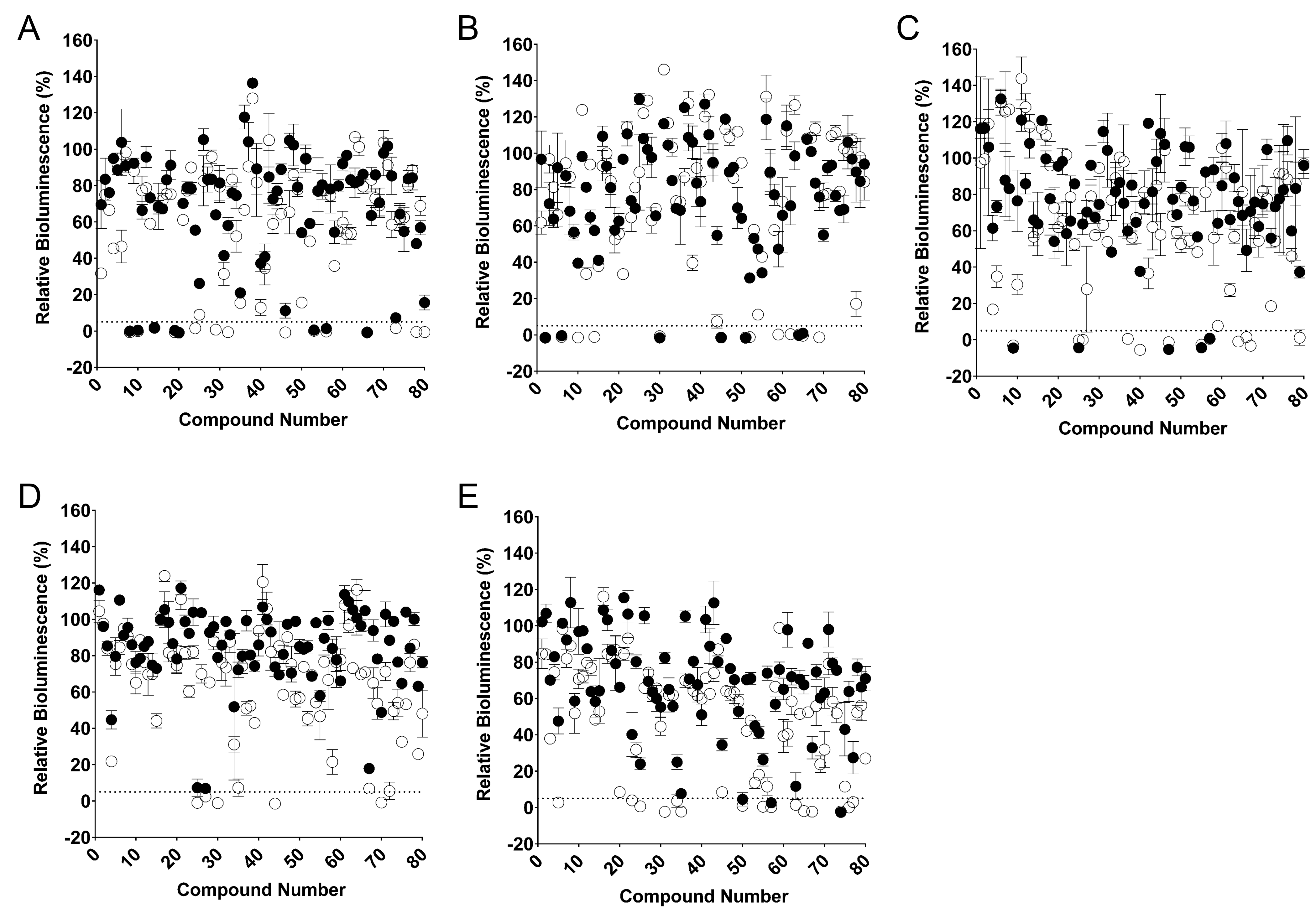

Supplement: S7 Fig — The relative bioluminescence (%) of the L. mexicana expressing NanoLuc-PEST when screened against two compound concentrations: 2 μM (filled circle) and 10 μM (open circle). The MMV Pathogen Box contains five plates, with 80 compounds per plate. The data for each plate is provided as a graph labelled with the plate identifier. (A) Plate A. (B) Plate B. (C) Plate C. (D) Plate D. (E) Plate E. Dashed lines indicate a decrease in bioluminescence of 5%. Mean values are shown (n = 4) ± SD from two independent experiments. Also see S2 Table. (TIF) [file pntd.0006639.s007.tif]
