# Supplementary material for: Development of NanoLuc-PEST expressing Leishmania mexicana as a new drug discovery tool for axenic- and intramacrophage-based assays
Source: PLoS Negl Trop Dis. 2018 Jul 12;12(7):e0006639. doi: 10.1371/journal.pntd.0006639 (PMC6057649; doi:10.1371/journal.pntd.0006639)
Supplement: S1 Table — (DOCX) [file pntd.0006639.s008.docx]

**S1 Table. Oligonucleotide sequences for cloning and integration.**

| Name | Purpose | Sequence | Restriction Enzyme |
| --- | --- | --- | --- |
| NanoLuc-F | Amplification of NanoLuc and NanoLuc-PEST for pSSU-Neo cloning | 5ʹ ‑GTTGGTGGATCCACCATGGTCTTCACAC‑3ʹ | *Bam*HI |
| NanoLuc-R |  | 5ʹ ‑GCCCCGGTACCAGAGTCGCGGCCTTACG‑3ʹ | *Kpn*I |
| NanoLuc-PEST-R |  | 5’- GCCCCGGTACCAGAGTCGCGGCCTTAG-3’ | *Kpn*I |
| PRE9-F | Amplification of PRE9 for pSSU-Neo cloning | 5ʹ ‑CTCACTATAGGGGATCCGCCCACCATGG‑3ʹ | *Bam*HI |
| PRE9-R |  | 5ʹ ‑GATTTTGGTACCGCTCATCACATCTTGGC‑3ʹ | *Kpn*I |
| pSSU-F | Validating integration into rDNA locus | 5’-TGTCAGATGTGCTGCTGCT-3’ | NA |
| pSSU-R |  | 5’-GCACCGACAAGAGACAGTTG-3’ | NA |
