# Supplementary material for: Development of NanoLuc-PEST expressing Leishmania mexicana as a new drug discovery tool for axenic- and intramacrophage-based assays
Source: PLoS Negl Trop Dis. 2018 Jul 12;12(7):e0006639. doi: 10.1371/journal.pntd.0006639 (PMC6057649; doi:10.1371/journal.pntd.0006639)
Supplement: S2 Table — Red values indicate the most potent compounds at 2 μM, blue values indicate the least potent compounds at 2 μM. (DOCX) [file pntd.0006639.s009.docx]

**S2 Table. Relative bioluminescence (%) following the MMV Pathogen Box screen at two compound concentrations (2 µM and 10 µM), against axenic amastigotes expressing NanoLuc-PEST. Red values indicate the most potent compounds at 2 µM, blue values indicate the least potent compounds at 2 µM.**

|  |  |  | Relative Bioluminescence (%) | |
| --- | --- | --- | --- | --- |
| Coumpound ID | Disease Set | Common Name | 10 µM | 2 µM |
| *MMV690102* | Kinetoplastids |  | -1.34 | -5.29 |
| *MMV595321* | Kinetoplastids |  | -3.10 | -4.54 |
| *MMV687251* | Tuberculosis |  | -0.24 | -4.37 |
| *MMV688262* | Tuberculosis | Delamanid | -2.71 | -4.37 |
| *MMV688978* | Reference Compound | Auranofin | -2.20 | -2.60 |
| *MMV019189* | Malaria |  | -1.42 | -1.73 |
| *MMV688763* | Schistosomiasis |  | -0.71 | -1.63 |
| *MMV652003* | Kinetoplastids |  | -1.54 | -1.53 |
| *MMV002817* | Onchocerciasis | Iodoquinol | -1.46 | -1.49 |
| *MMV676477* | Tuberculosis |  | -0.92 | -0.98 |
| *MMV676558* | Tuberculosis |  | -0.77 | -0.86 |
| *MMV011903* | Malaria |  | -1.14 | -0.43 |
| *MMV689480* | Reference Compound | Buparvaquone | 0.15 | -0.24 |
| *MMV676501* | Tuberculosis |  | -0.69 | -0.04 |
| *MMV102872* | Tuberculosis |  | -0.65 | 0.33 |
| *MMV676412* | Tuberculosis |  | -0.09 | 0.46 |
| *MMV676388* | Tuberculosis |  | 0.16 | 0.56 |
| *MMV687807* | Tuberculosis |  | 0.53 | 0.88 |
| *MMV003152* | Reference Compound | Mebendazole | -0.49 | 0.90 |
| *MMV676476* | Tuberculosis |  | -0.27 | 1.36 |
| *MMV688776* | Kinetoplastids |  | 1.70 | 1.90 |
| *MMV272144* | Tuberculosis |  | 0.19 | 2.64 |
| *MMV153413* | Tuberculosis |  | 0.97 | 4.50 |
| *MMV688467* | Kinetoplastids |  | 2.46 | 6.98 |
| *MMV099637* | Kinetoplastids |  | 1.60 | 7.25 |
| *MMV676162* | Kinetoplastids |  | -0.97 | 7.36 |
| *MMV001499* | Reference Compound | Nifurtimox | -2.17 | 7.65 |
| *MMV090930* | Tuberculosis |  | -0.77 | 11.16 |
| *MMV021013* | Tuberculosis |  | 1.47 | 11.73 |
| *MMV676512* | Tuberculosis |  | -0.58 | 15.66 |
| *MMV688372* | Kinetoplastids |  | 6.87 | 17.89 |
| *MMV688942* | Kinetoplastids | Bitertanol | 15.45 | 20.98 |
| *MMV689244* | Kinetoplastids |  | 0.66 | 23.97 |
| *MMV028694* | Malaria |  | 3.77 | 24.99 |
| *MMV688943* | Kinetoplastids | Difenoconazol | 8.96 | 26.19 |
| *MMV688755* | Tuberculosis |  | 0.45 | 26.38 |
| *MMV393995* | Tuberculosis |  | 3.00 | 27.43 |
| *MMV688774* | Reference Compound | Posaconazole | -1.25 | 31.32 |
| *MMV658988* | Kinetoplastids |  | -2.26 | 32.93 |
| *MMV688853* | Cryptosporidiosis |  | 42.96 | 34.19 |
| *MMV689243* | Kinetoplastids |  | 8.50 | 34.53 |
| *MMV689437* | Kinetoplastids |  | 1.17 | 37.21 |
| *MMV676409* | Tuberculosis |  | 12.84 | 37.25 |
| *MMV1030799* | Malaria |  | -5.56 | 37.62 |
| *MMV688761* | Schistosomiasis |  | -1.44 | 39.52 |
| *MMV671636* | Onchocerciasis |  | 3.97 | 40.19 |
| *MMV688514* | Kinetoplastids |  | 34.58 | 40.80 |
| *MMV687800* | Reference Compound | Clofazimine | 37.90 | 41.07 |
| *MMV676270* | Malaria |  | 18.00 | 41.26 |
| *MMV687762* | Kinetoplastids |  | 31.40 | 41.49 |
| *MMV688990* | Reference Compound | Miltefosine | 11.50 | 43.04 |
| *MMV010576* | Malaria |  | 21.84 | 44.69 |
| *MMV016838* | Malaria |  | 13.69 | 44.83 |
| *MMV020320* | Malaria |  | 0.21 | 47.21 |
| *MMV637229* | Trichuriasis | Clemastine | 11.15 | 47.31 |
| *MMV688775* | Reference Compound | Rifampicin | 2.74 | 47.59 |
| *MMV461553* | Tuberculosis |  | -0.44 | 48.03 |
| *MMV687703* | Tuberculosis |  | 76.96 | 48.25 |
| *MMV676411* | Tuberculosis |  | -0.81 | 48.75 |
| *MMV687180* | Tuberculosis |  | 1.44 | 49.23 |
| *MMV676384* | Tuberculosis |  | 59.90 | 51.03 |
| *MMV675995* | Onchocerciasis |  | 31.11 | 51.88 |
| *MMV687813* | Tuberculosis |  | 58.34 | 52.93 |
| *MMV003270* | Hookworm | Zoxazolamine | 57.93 | 53.16 |
| *MMV676589* | Tuberculosis |  | 15.64 | 54.08 |
| *MMV689028* | Kinetoplastids |  | 72.62 | 54.14 |
| *MMV676406* | Tuberculosis |  | 35.79 | 54.35 |
| *MMV687775* | Lymphatic Filariasis |  | 7.40 | 54.77 |
| *MMV676539* | Tuberculosis |  | 62.48 | 54.78 |
| *MMV000023* | Reference Compound | Primaquine | 76.84 | 55.01 |
| *MMV611037* | Tuberculosis |  | 44.60 | 55.30 |
| *MMV690028* | Kinetoplastids |  | 1.50 | 55.46 |
| *MMV667494* | Malaria |  | 61.89 | 55.75 |
| *MMV688313* | Schistosomiasis |  | 18.47 | 55.91 |
| *MMV020512* | Malaria |  | 56.11 | 56.59 |
| *MMV687138* | Tuberculosis |  | 48.25 | 56.61 |
| *MMV676520* | Tuberculosis |  | 68.82 | 56.86 |
| *MMV026313* | Malaria |  | 66.48 | 56.90 |
| *MMV687776* | Lymphatic Filariasis |  | -1.15 | 57.43 |
| *MMV007638* | Malaria |  | 52.58 | 57.66 |
| *MMV023985* | Malaria |  | 46.63 | 57.95 |
| *MMV1028806* | Malaria |  | -0.60 | 58.01 |
| *MMV689000* | Reference Compound | Amphotericin B | 48.35 | 58.31 |
| *MMV688283* | Kinetoplastids |  | 101.72 | 58.40 |
| *MMV688270* | Schistosomiasis |  | 51.88 | 58.63 |
| *MMV688471* | Toxoplasmosis |  | 49.33 | 59.14 |
| *MMV690103* | Kinetoplastids |  | 0.48 | 59.86 |
| *MMV1019989* | Malaria |  | 45.92 | 59.90 |
| *MMV676524* | Tuberculosis |  | 65.52 | 60.00 |
| *MMV676492* | Lymphatic Filariasis |  | 23.83 | 60.28 |
| *MMV688122* | Tuberculosis |  | 16.72 | 61.39 |
| *MMV023370* | Malaria |  | 54.43 | 62.35 |
| *MMV021057* | Malaria | Azoxystrobin | 55.66 | 62.63 |
| *MMV688415* | Kinetoplastids |  | 31.88 | 63.04 |
| *MMV675996* | Onchocerciasis |  | 25.83 | 63.13 |
| *MMV688555* | Tuberculosis |  | 83.23 | 63.56 |
| *MMV020165* | Malaria |  | 61.89 | 63.67 |
| *MMV687254* | Tuberculosis |  | -0.07 | 63.72 |
| *MMV006372* | Malaria |  | 81.31 | 63.77 |
| *MMV688846* | Tuberculosis |  | 60.58 | 63.77 |
| *MMV688938* | Tuberculosis |  | 76.80 | 63.80 |
| *MMV688273* | Kinetoplastids |  | 0.09 | 63.86 |
| *MMV676395* | Tuberculosis |  | 0.76 | 63.89 |
| *MMV004168* | Kinetoplastids |  | 52.89 | 64.14 |
| *MMV062221* | Malaria |  | 7.64 | 64.16 |
| *MMV560185* | Malaria |  | 94.77 | 64.27 |
| *MMV688798* | Kinetoplastids |  | 62.11 | 64.37 |
| *MMV688845* | Tuberculosis |  | 67.13 | 64.60 |
| *MMV024443* | Malaria |  | 32.60 | 64.75 |
| *MMV002529* | Reference Compound | Praziquantel | 93.76 | 64.85 |
| *MMV200748* | Tuberculosis |  | 61.91 | 64.99 |
| *MMV688543* | Dengue |  | 39.32 | 65.10 |
| *MMV687243* | Tuberculosis |  | 78.50 | 65.34 |
| *MMV019721* | Malaria |  | 69.43 | 65.53 |
| *MMV085210* | Malaria |  | 73.33 | 65.83 |
| *MMV687749* | Tuberculosis |  | 56.67 | 66.01 |
| *MMV687812* | Tuberculosis |  | 77.79 | 66.08 |
| *MMV688352* | Dengue |  | 8.52 | 66.17 |
| *MMV675993* | Cryptosporidiosis |  | 27.37 | 66.21 |
| *MMV688550* | Kinetoplastids |  | 56.13 | 66.32 |
| *MMV1110498* | Wolbachia LF |  | 77.35 | 66.34 |
| *MMV676389* | Tuberculosis |  | 71.87 | 67.21 |
| *MMV689029* | Kinetoplastids |  | 57.59 | 67.41 |
| *MMV687700* | Tuberculosis |  | 61.64 | 67.55 |
| *MMV688754* | Kinetoplastids | Trifloxystrobin | -1.92 | 67.57 |
| *MMV020623* | Malaria |  | 86.96 | 68.14 |
| *MMV668727* | Onchocerciasis |  | 78.89 | 68.46 |
| *MMV687273* | Tuberculosis |  | 81.32 | 68.48 |
| *MMV688934* | Kinetoplastids | Tolfenpyrad | 68.13 | 68.55 |
| *MMV689255* | Cryptosporidiosis | D-Eritadenine | 73.04 | 68.65 |
| *MMV024937* | Malaria |  | 69.28 | 68.74 |
| *MMV021375* | Malaria |  | 58.94 | 68.90 |
| *MMV019742* | Malaria |  | 102.67 | 69.14 |
| *MMV676260* | Malaria |  | 85.22 | 69.43 |
| *MMV687765* | Tuberculosis |  | 74.28 | 69.43 |
| *MMV010764* | Malaria |  | 31.69 | 69.50 |
| *MMV000063* | Reference Compound | Sitamaquine | 73.62 | 69.63 |
| *MMV001625* | Reference Compound | α-Difluoromethylornithine | 81.23 | 69.72 |
| *MMV019807* | Malaria |  | 111.93 | 69.92 |
| *MMV688771* | Schistosomiasis |  | 37.89 | 70.08 |
| *MMV084603* | Malaria |  | 61.10 | 70.21 |
| *MMV688509* | Toxoplasmosis |  | 27.84 | 70.28 |
| *MMV019551* | Malaria |  | 42.20 | 70.29 |
| *MMV407834* | Malaria |  | 63.07 | 70.36 |
| *MMV688279* | Kinetoplastids |  | 75.33 | 70.45 |
| *MMV676588* | Tuberculosis |  | 69.13 | 70.58 |
| *MMV392832* | Malaria |  | 51.50 | 70.60 |
| *MMV1088520* | Malaria |  | -3.27 | 70.72 |
| *MMV010545* | Malaria |  | 68.48 | 70.73 |
| *MMV495543* | Tuberculosis |  | 27.02 | 70.90 |
| *MMV688552* | Schistosomiasis |  | 47.85 | 71.00 |
| *MMV688991* | Reference Compound | Nitazoxanide | 0.38 | 71.09 |
| *MMV688557* | Tuberculosis |  | 58.46 | 71.96 |
| *MMV000062* | Reference Compound | Pentamidine | 96.76 | 72.21 |
| *MMV688274* | Kinetoplastids |  | 7.31 | 72.23 |
| *MMV553002* | Tuberculosis |  | 58.91 | 72.57 |
| *MMV045105* | Kinetoplastids |  | 44.11 | 73.02 |
| *MMV688889* | Tuberculosis |  | 59.03 | 73.23 |
| *MMV687172* | Tuberculosis |  | 54.36 | 73.27 |
| *MMV688852* | Toxoplasmosis |  | 34.77 | 73.28 |
| *MMV688762* | Schistosomiasis |  | 84.34 | 73.33 |
| *MMV659004* | Kinetoplastids |  | -1.44 | 73.83 |
| *MMV676382* | Schistosomiasis |  | 64.75 | 73.92 |
| *MMV228911* | Tuberculosis |  | 11.53 | 73.96 |
| *MMV676050* | Cryptosporidiosis |  | 42.99 | 74.33 |
| *MMV022236* | Malaria |  | 94.61 | 74.51 |
| *MMV688793* | Kinetoplastids |  | 52.13 | 74.59 |
| *MMV084864* | Malaria |  | 55.81 | 74.64 |
| *MMV024829* | Malaria |  | 69.82 | 74.80 |
| *MMV688703* | Toxoplasmosis |  | 81.84 | 74.84 |
| *MMV675994* | Cryptosporidiosis |  | 81.16 | 74.96 |
| *MMV687188* | Tuberculosis |  | 98.10 | 75.17 |
| *MMV688939* | Tuberculosis |  | 51.85 | 75.51 |
| *MMV688891* | Tuberculosis |  | 64.36 | 75.74 |
| *MMV161996* | Tuberculosis |  | 98.90 | 75.91 |
| *MMV688768* | Schistosomiasis |  | -1.33 | 75.96 |
| *MMV661713* | Tuberculosis |  | 83.31 | 76.01 |
| *MMV688417* | Toxoplasmosis |  | -0.98 | 76.07 |
| *MMV688416* | Dengue |  | 66.54 | 76.11 |
| *MMV026356* | Malaria |  | 65.05 | 76.21 |
| *MMV688980* | Malaria |  | 48.08 | 76.34 |
| *MMV023969* | Tuberculosis |  | 74.16 | 76.39 |
| *MMV687747* | Tuberculosis |  | 30.38 | 76.47 |
| *MMV687729* | Tuberculosis |  | 62.67 | 76.47 |
| *MMV676881* | Malaria |  | 54.00 | 76.51 |
| *MMV687803* | Reference Compound | Linezolid | 111.44 | 76.55 |
| *MMV202553* | Kinetoplastids |  | 77.09 | 76.92 |
| *MMV688797* | Kinetoplastids |  | 71.92 | 76.99 |
| *MMV676528* | Malaria |  | 57.74 | 77.13 |
| *MMV1236379* | Kinetoplastids |  | 52.39 | 77.18 |
| *MMV689709* | Kinetoplastids |  | 68.10 | 77.39 |
| *MMV688844* | Tuberculosis |  | 91.56 | 77.54 |
| *MMV689061* | Kinetoplastids |  | 95.91 | 77.60 |
| *MMV688474* | Kinetoplastids |  | 76.43 | 77.71 |
| *MMV676377* | Tuberculosis |  | 74.59 | 78.14 |
| *MMV676182* | Cryptosporidiosis |  | 53.64 | 78.18 |
| *MMV688941* | Tuberculosis |  | 74.74 | 78.23 |
| *MMV688888* | Tuberculosis |  | 89.99 | 78.28 |
| *MMV011511* | Malaria |  | 88.55 | 78.49 |
| *MMV688548* | Toxoplasmosis |  | 77.20 | 78.88 |
| *MMV676008* | Kinetoplastids |  | -1.15 | 79.00 |
| *MMV688795* | Kinetoplastids |  | 86.70 | 79.12 |
| *MMV676445* | Tuberculosis |  | 77.96 | 79.21 |
| *MMV687796* | Reference Compound | Amikacin | 58.25 | 79.34 |
| *MMV032967* | Malaria |  | 75.35 | 79.58 |
| *MMV023860* | Malaria |  | 74.37 | 79.77 |
| *MMV676461* | Tuberculosis |  | 54.88 | 79.84 |
| *MMV676599* | Cryptosporidiosis |  | 31.83 | 80.19 |
| *MMV030734* | Malaria |  | 87.08 | 80.21 |
| *MMV023949* | Malaria |  | 52.45 | 80.37 |
| *MMV688936* | Tuberculosis |  | 77.21 | 80.40 |
| *MMV023227* | Malaria |  | 64.23 | 80.48 |
| *MMV688364* | Toxoplasmosis |  | 58.45 | 80.73 |
| *MMV676605* | Malaria |  | 84.63 | 81.06 |
| *MMV676604* | Kinetoplastids |  | 33.57 | 81.35 |
| *MMV676379* | Tuberculosis |  | 79.42 | 81.42 |
| *MMV687699* | Tuberculosis |  | 62.05 | 81.42 |
| *MMV188296* | Kinetoplastids |  | 106.72 | 81.46 |
| *MMV687248* | Tuberculosis |  | 88.99 | 81.54 |
| *MMV688766* | Schistosomiasis |  | -2.36 | 82.40 |
| *MMV1198433* | Schistosomiasis |  | 91.16 | 82.52 |
| *MMV688958* | Kinetoplastids |  | 101.30 | 82.55 |
| *MMV687798* | Reference Compound | Levofloxacin (-)-ofloxacin | 74.50 | 83.03 |
| *MMV676603* | Tuberculosis |  | 75.26 | 83.14 |
| *MMV008439* | Malaria |  | 126.81 | 83.20 |
| *MMV688704* | Toxoplasmosis |  | 53.57 | 83.23 |
| *MMV1037162* | Malaria |  | 85.96 | 83.26 |
| *MMV676584* | Tuberculosis |  | 89.36 | 83.30 |
| *MMV676439* | Tuberculosis |  | 95.86 | 83.45 |
| *MMV688472* | Toxoplasmosis |  | 74.96 | 83.48 |
| *MMV016136* | Malaria |  | 91.85 | 83.56 |
| *MMV006741* | Malaria |  | 113.49 | 83.56 |
| *MMV009135* | Malaria |  | 73.86 | 83.57 |
| *MMV202458* | Tuberculosis |  | 79.97 | 83.71 |
| *MMV676186* | Kinetoplastids |  | 21.54 | 83.95 |
| *MMV1029203* | Malaria |  | 52.70 | 83.99 |
| *MMV023388* | Malaria |  | 76.22 | 84.10 |
| *MMV676474* | Tuberculosis |  | 88.48 | 84.28 |
| *MMV676380* | Malaria |  | 97.79 | 84.54 |
| *MMV676350* | Malaria |  | 104.94 | 84.72 |
| *MMV688921* | Dengue |  | 105.56 | 84.76 |
| *MMV011765* | Malaria |  | 45.24 | 84.80 |
| *MMV676536* | Schistosomiasis |  | 103.25 | 84.93 |
| *MMV019790* | Malaria |  | 56.75 | 85.04 |
| *MMV007625* | Malaria |  | 76.47 | 85.10 |
| *MMV688124* | Tuberculosis |  | 56.21 | 85.13 |
| *MMV688360* | Kinetoplastids |  | 58.50 | 85.35 |
| *MMV023953* | Malaria |  | 83.96 | 85.42 |
| *MMV676269* | Malaria |  | 76.17 | 85.78 |
| *MMV687730* | Tuberculosis |  | 52.52 | 85.81 |
| *MMV688547* | Kinetoplastids |  | 128.07 | 85.85 |
| *MMV676597* | Tuberculosis |  | 73.07 | 85.91 |
| *MMV024406* | Malaria |  | 93.72 | 85.94 |
| *MMV687706* | Kinetoplastids |  | 75.35 | 85.99 |
| *MMV063404* | Tuberculosis |  | 79.51 | 86.21 |
| *MMV047015* | Tuberculosis |  | 82.90 | 86.47 |
| *MMV688125* | Tuberculosis |  | 100.33 | 86.53 |
| *MMV024035* | Malaria |  | 79.61 | 86.60 |
| *MMV676470* | Tuberculosis |  | 79.72 | 87.35 |
| *MMV020591* | Malaria |  | 94.51 | 87.56 |
| *MMV007471* | Malaria |  | 69.68 | 87.80 |
| *MMV688327* | Tuberculosis | Radezolid | 125.56 | 87.81 |
| *MMV022478* | Malaria |  | 5.54 | 88.45 |
| *MMV688796* | Kinetoplastids |  | 91.91 | 88.65 |
| *MMV002816* | Reference Compound | Diethylcarbamazine | 62.54 | 88.75 |
| *MMV688756* | Tuberculosis | Sutezolid | 64.54 | 88.90 |
| *MMV021660* | Tuberculosis |  | 56.67 | 89.16 |
| *MMV676444* | Tuberculosis |  | 81.76 | 89.21 |
| *MMV019087* | Malaria |  | 89.32 | 89.61 |
| *MMV024195* | Malaria |  | 76.61 | 89.61 |
| *MMV020152* | Malaria |  | 108.81 | 89.71 |
| *MMV020391* | Malaria |  | 17.09 | 89.71 |
| *MMV001561* | Kinetoplastids | Fluoxetine | 52.43 | 90.42 |
| *MMV688553* | Tuberculosis |  | 98.31 | 90.94 |
| *MMV676401* | Tuberculosis |  | 75.18 | 91.23 |
| *MMV688178* | Schistosomiasis |  | 94.95 | 91.24 |
| *MMV026550* | Malaria |  | 87.66 | 91.50 |
| *MMV676602* | Kinetoplastids |  | 77.62 | 91.95 |
| *MMV676509* | Tuberculosis |  | 59.65 | 92.05 |
| *MMV688854* | Cryptosporidiosis |  | 66.13 | 92.07 |
| *MMV024397* | Malaria |  | 86.39 | 92.24 |
| *MMV393144* | Malaria |  | 81.97 | 92.24 |
| *MMV676449* | Tuberculosis |  | 88.05 | 92.26 |
| *MMV026490* | Malaria |  | 60.31 | 92.31 |
| *MMV687189* | Tuberculosis |  | 81.16 | 92.36 |
| *MMV675998* | Kinetoplastids |  | 65.21 | 92.81 |
| *MMV020120* | Malaria |  | 99.97 | 92.92 |
| *MMV676358* | Malaria |  | 64.12 | 93.00 |
| *MMV085071* | Malaria |  | 82.11 | 93.07 |
| *MMV000014* | Reference Compound | Mefloquine | 109.49 | 93.41 |
| *MMV676478* | Tuberculosis |  | 56.18 | 93.56 |
| *MMV658993* | Kinetoplastids |  | 64.93 | 93.82 |
| *MMV688994* | Reference Compound | Streptomycin | 84.34 | 94.02 |
| *MMV026020* | Malaria |  | 94.82 | 94.58 |
| *MMV689758* | Reference Compound | Bedaquiline | 45.41 | 94.89 |
| *MMV000011* | Reference Compound | Doxycycline | 94.68 | 94.97 |
| *MMV688362* | Kinetoplastids |  | 90.68 | 95.50 |
| *MMV688371* | Kinetoplastids |  | 61.94 | 95.57 |
| *MMV000907* | Malaria |  | 78.62 | 95.69 |
| *MMV659010* | Kinetoplastids |  | 88.01 | 95.95 |
| *MMV688955* | Toxoplasmosis |  | 96.76 | 95.96 |
| *MMV688361* | Kinetoplastids |  | 78.94 | 96.04 |
| *MMV020670* | Malaria |  | 97.66 | 96.09 |
| *MMV663250* | Malaria |  | 69.77 | 96.52 |
| *MMV688470* | Toxoplasmosis |  | 53.17 | 96.68 |
| *MMV690027* | Kinetoplastids |  | 33.47 | 96.72 |
| *MMV676480* | Onchocerciasis |  | 61.83 | 96.73 |
| *MMV006901* | Malaria |  | 107.15 | 96.82 |
| *MMV019993* | Malaria |  | 70.73 | 96.83 |
| *MMV687794* | Malaria |  | 72.05 | 97.30 |
| *MMV032995* | Malaria |  | 90.22 | 97.31 |
| *MMV020517* | Malaria |  | 63.10 | 97.70 |
| *MMV146306* | Tuberculosis |  | 40.43 | 97.77 |
| *MMV676554* | Tuberculosis |  | 103.74 | 97.83 |
| *MMV688330* | Toxoplasmosis |  | 79.07 | 97.96 |
| *MMV687146* | Tuberculosis |  | 84.77 | 98.04 |
| *MMV085499* | Malaria |  | 54.27 | 98.08 |
| *MMV688508* | Tuberculosis |  | 65.29 | 98.16 |
| *MMV012074* | Tuberculosis |  | 123.86 | 98.27 |
| *MMV688180* | Kinetoplastids |  | 81.67 | 98.32 |
| *MMV687801* | Reference Compound | Ethambutol | 126.55 | 98.57 |
| *MMV006833* | Malaria |  | 81.43 | 98.68 |
| *MMV020081* | Malaria |  | 73.47 | 98.78 |
| *MMV688271* | Kinetoplastids |  | 56.10 | 98.92 |
| *MMV024101* | Malaria |  | 49.51 | 98.92 |
| *MMV688407* | Kinetoplastids |  | 50.94 | 99.20 |
| *MMV676063* | Onchocerciasis |  | 66.65 | 99.42 |
| *MMV689060* | Kinetoplastids |  | 112.63 | 99.56 |
| *MMV022029* | Malaria |  | 101.70 | 99.81 |
| *MMV085230* | Malaria |  | 105.91 | 100.02 |
| *MMV675968* | Cryptosporidiosis |  | 86.04 | 100.09 |
| *MMV676877* | Malaria |  | 116.30 | 100.77 |
| *MMV000858* | Malaria |  | 108.52 | 100.91 |
| *MMV676159* | Kinetoplastids |  | 98.27 | 101.47 |
| *MMV688350* | Dengue |  | 91.24 | 101.75 |
| *MMV020710* | Malaria |  | 128.98 | 102.08 |
| *MMV011229* | Malaria |  | 84.66 | 102.18 |
| *MMV676571* | Tuberculosis |  | 85.94 | 102.29 |
| *MMV007133* | Malaria |  | 71.20 | 102.77 |
| *MMV023183* | Malaria |  | 84.53 | 103.26 |
| *MMV020289* | Malaria |  | 71.21 | 103.50 |
| *MMV024114* | Malaria |  | 69.99 | 103.63 |
| *MMV676526* | Tuberculosis |  | 46.48 | 103.78 |
| *MMV687246* | Malaria |  | 82.04 | 103.95 |
| *MMV688469* | Toxoplasmosis |  | 53.28 | 103.98 |
| *MMV676555* | Tuberculosis |  | 90.53 | 104.08 |
| *MMV676048* | Kinetoplastids |  | 53.90 | 104.27 |
| *MMV637953* | Reference Compound | Suramin | 116.61 | 104.52 |
| *MMV675969* | Onchocerciasis |  | 77.56 | 104.76 |
| *MMV407539* | Wolbachia LF |  | 70.84 | 104.77 |
| *MMV676431* | Tuberculosis |  | 65.31 | 104.86 |
| *MMV688345* | Toxoplasmosis |  | 69.94 | 105.25 |
| *MMV011691* | Malaria |  | 73.17 | 105.27 |
| *MMV053220* | Tuberculosis |  | 82.56 | 105.30 |
| *MMV676064* | Onchocerciasis |  | 123.90 | 105.34 |
| *MMV688411* | Toxoplasmosis |  | 65.85 | 105.51 |
| *MMV688179* | Kinetoplastids |  | 54.50 | 105.96 |
| *MMV687239* | Tuberculosis |  | 118.80 | 106.04 |
| *MMV019234* | Malaria |  | 39.61 | 106.08 |
| *MMV009054* | Malaria |  | 100.62 | 106.08 |
| *MMV676053* | Cryptosporidiosis |  | 78.76 | 106.26 |
| *MMV676472* | Tuberculosis |  | 93.04 | 106.46 |
| *MMV023233* | Malaria |  | 120.49 | 106.85 |
| *MMV676468* | Tuberculosis |  | 84.33 | 106.85 |
| *MMV687170* | Tuberculosis |  | 104.36 | 107.56 |
| *MMV006239* | Malaria |  | 108.03 | 107.64 |
| *MMV676191* | Cryptosporidiosis |  | 94.76 | 107.96 |
| *MMV020136* | Malaria |  | 122.06 | 108.03 |
| *MMV688466* | Tuberculosis |  | 117.26 | 108.11 |
| *MMV676161* | Kinetoplastids |  | 116.07 | 108.65 |
| *MMV020520* | Malaria |  | 127.42 | 108.71 |
| *MMV020982* | Malaria |  | 93.07 | 109.38 |
| *MMV024311* | Tuberculosis |  | 88.36 | 109.56 |
| *MMV001059* | Malaria |  | 96.75 | 109.79 |
| *MMV688773* | Reference Compound | Benznidazole | 132.20 | 110.17 |
| *MMV031011* | Malaria |  | 84.55 | 110.57 |
| *MMV676600* | Kinetoplastids |  | 114.36 | 110.66 |
| *MMV634140* | Malaria |  | 73.96 | 112.61 |
| *MMV007920* | Malaria |  | 88.96 | 112.76 |
| *MMV687696* | Tuberculosis |  | 57.87 | 113.56 |
| *MMV007803* | Malaria |  | 107.95 | 113.68 |
| *MMV688410* | Kinetoplastids |  | 63.15 | 114.60 |
| *MMV069458* | Tuberculosis |  | 112.41 | 115.05 |
| *MMV676398* | Wolbachia LF |  | 84.49 | 115.47 |
| *MMV026468* | Malaria |  | 104.46 | 116.14 |
| *MMV675997* | Kinetoplastids |  | 97.48 | 116.18 |
| *MMV020537* | Malaria |  | 146.04 | 116.22 |
| *MMV676204* | Onchocerciasis |  | 99.37 | 116.49 |
| *MMV020291* | Malaria |  | 111.14 | 117.25 |
| *MMV688554* | Tuberculosis |  | 66.57 | 117.68 |
| *MMV020321* | Malaria |  | 131.13 | 118.65 |
| *MMV676442* | Malaria |  | 113.29 | 118.75 |
| *MMV676057* | Kinetoplastids |  | 36.52 | 119.16 |
| *MMV054312* | Tuberculosis |  | 116.16 | 120.77 |
| *MMV020388* | Malaria |  | 143.84 | 121.02 |
| *MMV019838* | Malaria |  | 86.78 | 125.18 |
| *MMV676386* | Tuberculosis |  | 120.21 | 127.03 |
| *MMV001493* | Onchocerciasis | Isradipine | 89.57 | 129.79 |
| *MMV687145* | Tuberculosis |  | 130.77 | 132.61 |
| *MMV676383* | Tuberculosis |  | 127.92 | 136.33 |
