# Supplementary material for: Development of NanoLuc-PEST expressing Leishmania mexicana as a new drug discovery tool for axenic- and intramacrophage-based assays
Source: PLoS Negl Trop Dis. 2018 Jul 12;12(7):e0006639. doi: 10.1371/journal.pntd.0006639 (PMC6057649; doi:10.1371/journal.pntd.0006639)
Supplement: S3 Table — EC50 values are colour-coded, where red indicates the most potent compounds, and blue indicates the least potent compounds. (DOCX) [file pntd.0006639.s010.docx]

**S3 Table. EC_50_ values for selected ‘hit’ compounds, from the axenic amastigote screen. EC_50_ values are colour-coded, where red indicates the most potent compounds, and blue indicates the least potent compounds.**

|  |  |  | Axenic Amastigotes | |
| --- | --- | --- | --- | --- |
| Compound ID | MMV Disease Set | Common Name | EC_50_ (µM) | 95% Confidence Intervals |
| *MMV689480* | Reference Compound | Buparvaquone | 0.0022 | 0.0017-0.002 |
| *MMV688262* | Tuberculosis | Delamanid | 0.03 | 0.031-0.031 |
| *MMV690102* | Kinetoplastids |  | 0.06 | 0.038-0.054 |
| *MMV676477* | Tuberculosis |  | 0.07 | 0-06-0.072 |
| *MMV652003* | Kinetoplastids |  | 0.07 | 0.048-0.095 |
| *MMV595321* | Kinetoplastids |  | 0.15 | 0.13-0.25 |
| *MMV688978* | Reference Compound | Auranofin | 0.17 | 0.16-0.18 |
| *MMV011903* | Malaria |  | 0.18 | 0.12-0.15 |
| *MMV002817* | Onchocerciasis | Iodoquinol | 0.22 | 0.21-0.23 |
| *MMV687807* | Tuberculosis |  | 0.25 | 0.18-0.24 |
| *MMV676412* | Tuberculosis |  | 0.26 | 0.19-0.28 |
| *MMV676501* | Tuberculosis |  | 0.3 | 0.26-0.34 |
| *MMV019189* | Malaria |  | 0.32 | 0.29-0.40 |
| *MMV688763* | Schistosomiasis |  | 0.38 | 0.39 -0.48 |
| *MMV676558* | Tuberculosis |  | 0.47 | 0.37-0.49 |
| *MMV687251* | Tuberculosis |  | 0.48 | 0.48-0.49 |
| *MMV153413* | Tuberculosis |  | 0.52 | 0.45-0.57 |
| *MMV676476* | Tuberculosis |  | 0.58 | 0.53-0.73 |
| *MMV272144* | Tuberculosis |  | 0.64 | 0.59-0.87 |
| *MMV676388* | Tuberculosis |  | 0.73 | 0.54-0.69 |
| *MMV102872* | Tuberculosis |  | 0.81 | 0.80-1.44 |
| *MMV688776* | Kinetoplastids |  | 0.9 | 0.86-0.95 |
| *MMV676162* | Kinetoplastids |  | 0.96 | 0.80-1.02 |
| *MMV688467* | Kinetoplastids |  | 1.65 | 1.56-2.30 |
| *MMV003152* | Reference Compound | Mebendazole |  | |
