# Supplementary material for: Development of NanoLuc-PEST expressing Leishmania mexicana as a new drug discovery tool for axenic- and intramacrophage-based assays
Source: PLoS Negl Trop Dis. 2018 Jul 12;12(7):e0006639. doi: 10.1371/journal.pntd.0006639 (PMC6057649; doi:10.1371/journal.pntd.0006639)
Supplement: S4 Table — Data is obtained either from the MMV Pathogen Box website (a), or experimentally (b). (DOCX) [file pntd.0006639.s011.docx]

**S4 Table. Cytotoxicity data for selected ‘hit’ compounds. Data is obtained either from the MMV Pathogen Box website, or experimentally.**

| **MMV ID** | **HepG2 CC_20_ (μM)^a^** | **HepG2 CC_50_ (μM)^a^** | **MRC5 CC_50_ (μM)^a^** | **THP1 EC_50_ (µM)^b^** |
| --- | --- | --- | --- | --- |
| *MMV011903* | 8.4 | >10 | ND | >50 |
| *MMV676477* | ND | 1.3 | ND | >50 |
| *MMV595321* | ND | ND | ND | >50 |
| *MMV689480* | Reference Compound | | | |
| *MMV688262* | ND | 72.5 | ND | ND |
| *MMV652003* | 0.4 | ND | >32 | ND |
| *MMV690102* | ND | ND | 5.4 | ND |
| *MMV003152* | Reference Compound | | | |

^a^ Data provided with the MMV Pathogen Box.

^b^ Data obtained as part of this study.

ND = no data available.
